# Clinical, morphologic and molecular heterogeneity of HPV-associated oropharyngeal cancer

**DOI:** 10.1038/s41388-023-02819-y

**Published:** 2023-09-04

**Authors:** Yvonne X. Lim, Michelle L. Mierzwa, Maureen A. Sartor, Nisha J. D’Silva

**Affiliations:** 1 Periodontics and Oral Medicine, University of Michigan School of Dentistry, 1011N. University Ave, Ann Arbor, MI USA; 2 Rogel Cancer Center, University of Michigan, 1500 E Medical Center Dr, Ann Arbor, MI USA; 3 Department of Radiation Oncology, University of Michigan Medical School, Ann Arbor, MI USA; 4 Department of Computational Medicine and Bioinformatics, University of Michigan Medical School, Ann Arbor, MI USA; 5 Department of Biostatistics, School of Public Health, University of Michigan Medical School, Ann Arbor, MI USA; 6 Pathology, University of Michigan Medical School, Ann Arbor, MI USA

**Keywords:** Head and neck cancer, Biomarkers, Tumour virus infections, Tumour heterogeneity, Tumour biomarkers

## Abstract

The incidence of human papillomavirus-positive (HPV+) oropharyngeal squamous cell carcinoma (OPSCC) is rising rapidly and has exceeded cervical cancer to become the most common HPV-induced cancer in developed countries. Since patients with HPV + OPSCC respond very favorably to standard aggressive treatment, the emphasis has changed to reducing treatment intensity. However, recent multi-center clinical trials failed to show non-inferiority of de-escalation strategies on a population basis, highlighting the need to select low-risk patients likely to respond to de-intensified treatments. In contrast, there is a substantial proportion of patients who develop recurrent disease despite aggressive therapy. This supports that HPV + OPSCC is not a homogeneous disease, but comprises distinct subtypes with clinical and biological variations. The overall goal for this review is to identify biomarkers for HPV + OPSCC that may be relevant for patient stratification for personalized treatment. We discuss HPV + OPSCC as a heterogeneous disease from multifaceted perspectives including clinical behavior, tumor morphology, and molecular phenotype. Molecular profiling from bulk tumors as well as single-cell sequencing data are discussed as potential driving factors of heterogeneity between tumor subgroups. Finally, we evaluate key challenges that may impede in-depth investigations of HPV + OPSCC heterogeneity and outline potential future directions, including a section on racial and ethnic differences.

## Introduction

Oropharyngeal squamous cell carcinoma (OPSCC) represents a subset of head and neck squamous cell carcinomas (HNSCC) comprising malignancies in the tonsils, base of tongue, soft palate, and posterior pharyngeal wall. The global incidence of OPSCC has escalated in high-income and developed countries [1–3], which is primarily due to the rise in human papillomavirus (HPV) infection [3–5]. OPSCC has already overtaken cervical cancer to become the most common HPV-associated (HPV+) cancer in the United States and United Kingdom [1, 4, 6]. The 8th edition of the American Joint Committee on Cancer (AJCC-8) recently recognized HPV + OPSCC as a separate entity from classical, tobacco-associated HPV-negative [HPV(-)] OPSCC) [7, 8] with distinct demographics [1], clinical features [9, 10] and molecular profiles [11–13]. Importantly, patients with HPV + OPSCC have better survival due to favorable response to treatment [9, 10]. Similar to other HPV-related cancers, HPV E6 and E7 oncoproteins are key drivers for OPSCC largely due to inhibition of p53 and Rb, respectively [14, 15]. Clinically, p16 positivity by immunohistochemistry (IHC) is used to diagnose HPV-related malignancies with over 95% concordance to HPV DNA positivity [16]. However, discordance between HPV and p16 occurs in some patients with OPSCC who show poorer outcomes than those with HPV+ and p16+ OPSCC [17]. Additional confirmatory testing using in situ hybridization (ISH) or polymerase chain reaction (PCR) to detect HPV DNA or RNA is recommended [16].

Despite superior survival of HPV + OPSCC, standard treatment is associated with high toxicities and compromised quality-of-life [18, 19]. As a result, there is growing interest in de-escalating treatment for these patients. However, recently completed clinical trials to de-intensify chemoradiation in unselected populations failed to demonstrate non-inferiority [18, 20]. Moreover, a subset of HPV + OPSCC continues to progress despite standard aggressive treatment [10, 21]. These findings suggest heterogeneity among primary HPV + OPSCC that influences clinical outcomes. Here, we discuss evidence demonstrating dichotomy in primary HPV + OPSCC from clinical, morphological, and molecular perspectives. We also evaluate factors that may contribute to tumor heterogeneity, with the goal of summarizing biomarkers that could predict patient risk and optimize treatment selection.

## From oral HPV infection to OPSCC development

**Fig. 1:**
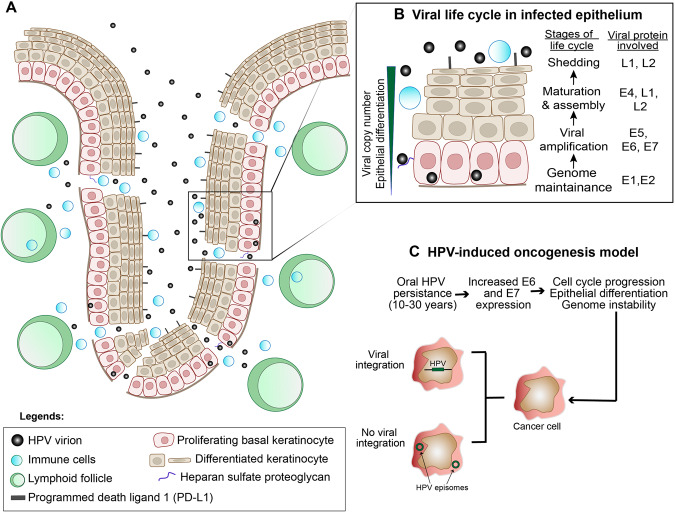
HPV life cycle and carcinogenesis in the tonsillar crypt. **A** Schematic diagram of an infected tonsillar crypt, a region that has high HPV predilection in the oropharynx. The reticulated epithelium, immune cell enrichment and high membranous PD-L1 expression are factors that foster HPV infection in the proliferating basal cells of the tonsillar crypt. HPV virion enters basal epithelial cells by binding heparan sulfate proteoglycan (**B**) HPV life cycle in infected stratified squamous epithelium. Stratified epithelium is enlarged from boxed region in (**A**). Progression of viral cycle is closely related to epithelial differentiation, and expression of various viral genes is upregulated at distinct stages. **C** HPV-induced oncogenesis model for OPSCC. Persistent HPV infection likely drives OPSCC formation through elevated E6 and E7 expression. Both E6 and E7 alter cellular functions such as cell cycle progression, epithelial differentiation, and genome instability that cause cancer. The HPV genome either integrates in host chromosomes or exists as circular episomes in cancer cells.

More than 200 types of HPV have been identified; 15 are high-risk and associated with cancer. Among these, HPV16 induces 80–90% of all HPV + OPSCC [22, 23]. Transmission of HPV to the oropharynx is through sexual contact [24] and infection normally occurs in basal and mitotically active epithelial cells. The productive life cycle of HPV is closely related to keratinocyte differentiation in stratified epithelium [25] that likely triggers extensive viral genome amplification, via a poorly understood mechanism [25] (Fig. 1). Most HPV infections are transient and cleared within 2 years [26–28]; failure to eliminate high-risk oral HPV may trigger neoplastic progression and malignancy [26–28]. Detection of high-risk HPV in oral samples has been shown to significantly increase the risk of OPSCC [29], and HPV oral DNA has been detected periodically for up to 7 years before OPSCC diagnosis [27, 29]. In contrast, seropositivity of HPV E6 could be detected 20–30 years before OPSCC [30]. Therefore, HPV infection likely occurs decades before clinical presentation. Within the oropharynx, HPV + OPSCC is highly distributed at the tonsillar crypt, which has reticulated epithelium that allows virions easy access to basal epithelial cells [31]. The basement membrane is also porous to immune cells that directly contact the basal epithelium (Fig. 1) [31]. Resident myeloid cells within the crypt may foster a permissive microenvironment that enables HPV to evade host immune surveillance [32, 33].

During persistent infection, E6 and E7 are highly expressed (Fig. 1), encouraging molecular alterations that shape the epithelial environment to initiate carcinogenesis through their key functions in suppressing p53 and Rb [6]. Other oncogenic functions of E6 and E7 include compromising cellular DNA repair, enhancing genomic instability, and increasing immune escape (Fig. 1) [34]. Increased E6 and E7 proteins may promote integration of HPV DNA into host genome, but only 50–70% of HPV+ OPSCCs harbor HPV integration [35–37]. It is likely that episomal HPV induces different carcinogenic mechanisms from integrated HPV [35]. Integration breakpoints and subsequent viral genome linearization may occur at the open reading frame of *E1*, *E2*, or *E5*, leading to their deletion or truncation and concomitant loss of expression [38]. As E2 is a transcriptional repressor for *E6/E7*, expression of E6 and E7 is enhanced [38]. However, recent studies showed that disruption of the *E2* gene does not necessarily follow viral integration [35, 39]. In OPSCC with integrated HPV but intact *E2*, methylation at the E2-binding sites in the upstream regulatory regions of *E6* and *E7* inhibited E2-mediated repression of *E6/E7* transcription, resulting in E6/E7 overexpression despite the presence of E2 [39].

Since HPV16 dominates HPV + OPSCC cases, most studies focus on HPV16 + OPSCC and assume that non-HPV16 genotypes behave similarly. Consequently, the exact prognostic impact of distinct HPV genotypes is unclear; some studies suggest that patients with non-HPV16 variants have inferior survival outcomes [40, 41], while others show no or minimal effect on survival [42, 43]. HPV16 is also more likely to infect the tonsillar crypt than the HPV33 variant [41], although the underlying molecular basis is not yet understood.

## Clinical management of HPV-associated OPSCC

**Fig. 2:**
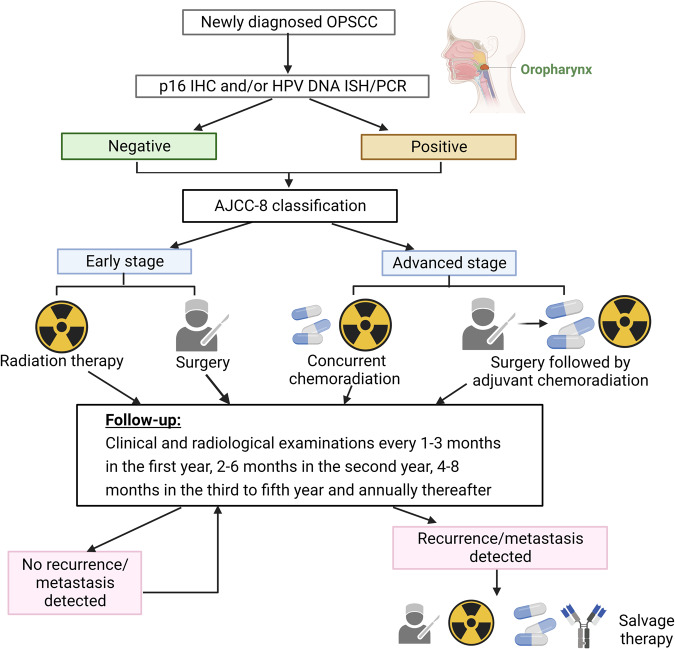
Treatment workflow for patients with OPSCC. HPV positivity is determined in newly diagnosed OPSCC using p16 immunohistochemistry (IHC) and/or HPV DNA in situ hybridization (ISH) or polymerase chain reaction (PCR). Tumors are staged according to AJCC-8 to determine the treatment plan. Upon completion, patients are followed up periodically. Recurrent or metastastic tumors are given salvage therapy, which may include surgery, radiation, chemo-, or immunotherapy. Created with BioRender.

Although HPV + OPSCC is associated with superior treatment outcomes, treatment recommendations for these patients remain the same as those for HPV(−) OPSCC. Early-stage patients receive surgery or radiation (RT), while patients with locally advanced cancer receive multimodal treatment, either concurrent chemotherapy and radiation (CRT) or surgery followed by adjuvant CRT [44] (Fig. 2). RT as definitive treatment is 70 Gy, while 60–66 Gy is recommended for adjuvant treatment [44]. Platinum-based agents such as cisplatin are usually given as chemotherapy [44, 45].

In the definitive setting, standard-of-care treatment often entails substantial morbidity [18, 20] and clinical trials focused on strategies to de-intensify standard therapies. These include: (i) substitution of cisplatin with cetuximab and immunotherapy in the standard CRT regimen; (ii) RT dose reduction to definitive and/or elective regions; (iii) adaptation of RT based on mid-treatment response; (iv) induction chemotherapy followed by de-intensified RT or CRT, and (v) transoral surgery (TORS) with de-escalated and risk-based adjuvant therapy (Supplementary Table 1). However, currently, no large multi-center phase III trial has proven non-inferiority of de-intensification paradigms in the definitive setting. There is a compelling need to identify true low-risk HPV + OPSCC likely to respond to de-escalated regimens.

Despite the good prognosis of primary HPV + OPSCC, locoregional recurrences and distant metastasis are observed [10, 21, 46]. Current guidelines recommend periodic clinical and radiological examinations for over 5 years to detect recurrence [47] (Fig. 2). Patients with recurrent tumors receive salvage therapy with curative intent such as salvage surgery, re-irradiation, or systemic chemotherapy [47] (Fig. 2). Salvage therapy significantly reduces risk of death in recurrent OPSCC by ~50%, with p16+ responding better than p16- tumors [21, 48]. Recently, PD-L1/PD-1 immune checkpoint inhibitors were approved by the U.S. Food and Drug Administration (FDA) to treat recurrent and metastatic HNSCC, regardless of HPV or p16 status [49]. Although p16+ HNSCC patients had superior responses to PD-L1/PD-1 inhibitors than their p16(−) counterparts, this was limited to recurrent and metastatic tumors [50–53]. In the definitive setting of primary and locally advanced HNSCC, PD-L1/PD-1 inhibitors did not provide benefit regardless of p16 status [54–56]. Since HPV E6 and E7 are constitutively expressed in HPV + OPSCC, they are attractive targets for therapeutic vaccines [57]. These therapeutic vaccines have yielded promising results in several Phase I and II clinical trials in locally advanced or recurrent and metastatic HPV + HNSCC, especially in conjunction with PD-L1/PD-1 therapy [58–60]. Further validation from prospective Phase III trials is needed for implementation into clinical routine.

In adoptive T-cell therapy, T cells extracted from a patient are engineered to target the tumor cells, expanded, and re-infused in the same patient. A recent Phase I clinical trial explored the effectiveness of T-cell receptor-engineered T cells targeting HPV E7 in 12 recurrent and metastatic HPV+ cancer patients [61]. Tumor regression was observed in 6 of 12 patients, suggesting high curative potential [61]. However, the patient cohort is small and comprises multiple cancer types, including cervical and anal cancers [61]. More clinical trials are needed to determine the effectiveness of T-cell therapy in locoregional HPV + OPSCC.

Although currently available therapies are effective for HPV + OPSCC at various stages (Fig. 2), responses vary due to inter-patient heterogeneity. Since de-escalation trials largely focus on primary HPV + OPSCC (Supplementary Table 1), this review focuses on tumor heterogeneity in primary HPV + OPSCC. This will allow emphasis on intrinsic characteristics associated with tumors that recur after initial treatment.

## Clinical heterogeneity of HPV-associated OPSCC

HPV status is a strong determinant of superior response to therapy, but prospective studies consistently demonstrated that a subset of patients (~25%) had disease progression within 2 years of treatment [9, 10]. This suggests that not all primary tumors respond favorably to standard aggressive therapy. To better understand differential clinical behaviors, Ang and colleagues performed recursive-partitioning analysis that classified all OPSCC into distinct risk groups [10]. Patients with HPV + OPSCC and tobacco exposure have an intermediate-risk phenotype with poorer survival than non-smokers. In contrast, HPV(−) OPSCC was classified in the high-risk group [10]. Several studies reported positive associations between tobacco exposure and survival of HPV + OPSCC, although controversies remain [62, 63]. Risk stratification analysis is essential for treatment de-escalation that aims to personalize therapeutic regimens. In two early clinical trials (RTOG 1016 and De-ESCALaTE), replacing cisplatin with cetuximab, a drug targeting EGFR, in the standard CRT regimen led to inferior survival in unselected HPV + OPSCC with no reduction in acute and long-term toxicities [18, 19]. In contrast, the FDA approval of TORS provides an alternative for radiation therapy de-escalation, as reflected by several de-intensification trials (Supplementary Table 1) but surgery has toxicity as well. Consequently, patient-reported outcomes have been numerically superior for definitive CRT strategies [64, 65]. Furthermore, some tumors still recur after TORS and survival rates after recurrence are low at around 60% [66]. To predict patients with lowest risk of recurrence, several de-escalation trials are selecting patients based on clinical, biologic, and imaging markers such as smoking history, extranodal extension, diffusion MRI, and FDG-PET metrics, and genomic alterations (Supplementary Table 1). Results from these clinical trials are awaited.

In summary, HPV + OPSCC demonstrate large variations in clinical behavior and treatment response. Results from completed and ongoing treatment de-intensification studies support the notion that patients with HPV + OPSCC require personalized treatment strategies. However, even with the AJCC-8 classification that better stratifies HPV + OPSCC than AJCC-7, there is no significant difference in overall survival between Stages I and II, or between Stages II and III patients [67, 68]. This suggests that clinical variables not included in AJCC-8 may significantly influence prognosis. Some of the proposed factors include extranodal extension [69–72], perineural invasion [73, 74], and angiolymphatic invasion [73], but controversies regarding their prognostic impact remain. Although p16 IHC is used to diagnose presence of HPV + OPSCC, a study shows that its cellular localization dictates survival outcomes [75, 76]. Understanding biological factors that implicate clinical dichotomy will refine cancer staging and better inform optimal therapeutic strategy for specific patient subgroups.

## Morphologic heterogeneity of HPV+OPSCC

Based on morphologic variations, there are three main subtypes: non-keratinizing, keratinizing, and non-keratinizing with maturation (NKM) [77]. These differ in keratinization, stromal reaction, and nuclear and cytoplasmic features among others (Table 1). About 50% of OPSCC show non-keratinizing morphology and most (70–98%) are HPV+ [78–80]. In contrast, keratinizing morphology is mostly associated with HPV(-) OPSCC, although a small fraction (~3–15%) of HPV + OPSCC also present this phenotype [78, 79, 81]. This suggests that a subset of HPV + OPSCC resembles the more aggressive HPV(−) cancer. In some keratinizing OPSCC, no true keratin formation is observed, but a dense layer of cytoplasmic eosinophilia imparted by keratin intermediate filaments is observed [77]. Together, these three subtypes comprise ~90% of OPSCC (Table 2), while the other 10% are rare variants such as basaloid, undifferentiated, and papillary carcinomas [80, 81].

**Table 1:** Characteristics of main morphologic subtypes of HPV + OPSCC.

Characteristics	Nonkeratinizing (NK)	Nonkeratinizing with maturation (NKM)	Keratinizing (K)	Ref.
Prevalence among both HPV+ and HPV(−) OPSCC	47–52.4% of all OPSCC.	11.3–23% of all OPSCC.	12–26.2% of all OPSCC.	[77–79, 81, 82]
HPV/p16 profile	70–100% are p16+ and/or HPV+	20–85% are p16+ and/or HPV+	20–35% are p16+ and/or HPV+	[78–81]
Prognosis/ tumor aggressivness	Best survival of the three subtypes.	Better survival than K, but worse than NK.	Worst survival of the three subtypes.	[78, 82]
Arrangment of tumor cells	Tumor cells are arranged haphazardly in large nests with distinct and smooth borders.	Mostly nonkeratinizing except for >10% of tumor area that shows mature squamous differentiation (i.e polygonal cells with mature, eosinophilic cytopasm, distinct cell borders, intracellular bridges and keration pearls). Squamous differentiation is located aberrantly at the periphery of the nests with artificial clefting.	Very large nests that are more irregular, haphazard, and angular than NK.	[77, 78]
Shape of tumor cells	Immature basal-like appearance with indistinct cell border.		Usually more oval or spindle than round, with prominent cell border.	[77, 78]
Degree of keratinization	Absence of keratinization.		Keratin formation is common. Dense cytoplasmic eosinophilia due to keratin intermediate filaments.	[77, 78]
Nuclear features	Hyperchromatic. Oval to spindle in shape and devoid of nucleoli.		N.A	[77, 78]
Cytoplasmic features	Loss of cytoplasmic border, syncytial cytoplasm. Small to modest amount of eosinophilic cytoplasm.		Abundant eosinophilic cytoplasm.	[77, 78]
Nuclear:cytoplasmic (N/C) ratio	High N/C ratio.		N.A	[77, 78]
Appearance of mitoses, apoptosis and necrosis	Excess mitiotic and apoptotic activity. Comdeo necrosis is typically observed.		N.A	[77, 78]
Anaplasa and multinucleation	Lowest of three subtypes. Usually only focal distribution.		Highest of three. Diffuse distribution.	[82]
Stomal content	Very little desmoplasia.		Prominent stromal desmoplasia.	[77, 78]

*N.A* not applicable.

**Table 2:** Published studies proposing molecular subtypes for HPV + OPSCC (References followed by an asterisk (*) can be consulted in the Supplementary References).

Authors (Year), Journal	Number of samples and HPV status	Number of samples by anatomic subsite	Platform	HPV detection method	Subtype names and features	Clinical outcomes	Comments	Ref.
TCGA (2015), Nature	36 HPV + , 243 HPV−	33 oropharynx (64% HPV + ) 246 non-oropharynx (6% HPV + )	RNA Seq	RNA Seq alignment	Basal (BA): Harbors *NOTCH1* inactivation with intact oxidative stress signaling; reduced *SOX2* expression, *HRAS–CASP8* co-mutation and 11q13/q22 co-amplifications. Mesenchymal (MS): Characterized by enriched immune response. Atypical (AT): Lacks Chr7 amplifications and is enriched for HPV+ tumors and activating *PIK3CA* mutations. Classical (CL): Contains *TP53* mutations, loss of function in *CDKN2A*, Chr3q ampliciation and alteration of oxidative stress and xenobiotic metabolism genes. Most have heavy smoking history and laryngeal tumors.	No significant differences were observed when both HPV+ and HPV (−) patients were included. However, when HPV+ patients were excluded, HPV(−) patients in AT subgroup had the worse prognosis.	Although most HPV+ were in atypical subgroup, a few were classified under classical/mesenchymal/basal subgroups.	[11]
Keck et al. (2015), Clinical Cancer Research	Training cohort:371 total including 134 from own cohort; 55 HPV+, 75 HPV(−), others undetermined. Validation cohort: 541 from TCGA and Affymetrix cohorts (470 HPV(−), 71 HPV+).	From own cohort: 29 larynx, 25 oral cavity, 75 oropharynx Unknown for other cohorts	Microarray	qPCR expression of HPV E6 and E7 or RNA Seq and exome alignment	Basal (BA): Made exclusively of HPV- HNSCC. Tumors are enriched for EGFR/neuregulin signaling and have high epithelial expression and increased keratinization. Amplification of *MYC* and *ITGB* is common. Classical (CL-HPV and CL-nonHPV): Both CL-HPV and CL-nonHPV are characterized by enriched putrescine degradation pathway, increased proliferation signature and increase amplification of *E2F3* and *PIK3CA*. CL-HPV is enriched in cell cycle genes while CL-nonHPV has increased xeonbiotic metabolism pathway expression. Inflamed/mesenchymal (IMS-HPV and IMS-nonHPV): Both IMS-HPV and IMS-nonHPV have high immune response characterized by CD8+ infiltration and enhanced EMT signature. IMS-HPV has lower keratination, enhanced cell cycle activities and are more poorly differentiated than CL-HPV.	IMS-HPV has higher 5-year survival than CL-HPV.	Oral cavity tumors are overrepresented in the basal group (72%, P = 1.04 × 10 − 5). About 40% of CL-HPV showed keratinization, but none of the IMS-HPV tumors is keratinizing.	[91]
Zhang et al. (2016), Clinical Cancer Research	84 HPV+, 18 HPV−	67 oropharynx (64 HPV + , 3 HPV) 30 oral cavity (17 HPV + , 13 HPV-) 3 larynx (1 HPV + , 2 HPV-) 2 hypopharynx (2 HPV-)	RNA Seq	Quantification of viral gene expression determined by RNA-Seq library	HPV-KRT: Increased keratinization/epidermal differentiation and oxidation-reduction processes; higher rate of HPV integration and lower expression of E2, E4 and E5 genes than HPV-IMU. Higher ratio of spliced E6 oncogene (E6^*^) relative to total E6 expression; increased amplifications on chr3q, higher copy number of PIK3CA and increased frequency of activating mutations. HPV-IMU: Elevated immune response and increased EMT signature compared to HPV-KRT. Frequent copy number loss on chr16q is observed.	HPV-KRT has a poorer overall survival compared to HPV-IMU.	This is the first study that correlates subgroups with HPV characteristics. Multidimensional scaling showed that KRT was overall more similar to HPV- samples.	[92]
Lee et al. (2018), Oral Oncology	Discovery cohort: 514 HNSCC, 95 ESCC, 485 LSCC and 252 CSCC from TCGA*. (37 HPV+, 72 HPV-, others undetermined) Validation cohort: 408 HNSCC patients (74 HPV+, 291 HPV−, others undetermined)	TCGA HNSCC cohort: 78 oropharynx, 302 oral cavity, 114 larynx, 9 hypopharynx Validation cohort: 136 oropharynx, 138 oral cavity, 78 larynx, 46 hypopharynx	RNA seq	RNA Seq alignment for TCGA cohort. In situ hybridization or E6 qPCR of HPV DNA and RNA in validation cohort	Gene expression profiles of HNSCC was analyzed and grouped according to their similarities to CSCC, LSCC and ESCC: Subtype 1: Similar to CSCC; enriched with PKA, VEGF, mTOR and IL8 signaling; least frequent *TP53* mutations Subtype 2: increased RhoA, PI3K/Akt and NFΚB signaling - fewest copy number alterations Subtype 3: - similar to LSCC - infrequent *PIK3CA* mutations compared to subtypes 1 and 3 - Enriched nicotine degradation, NOTCH, xenibiotic metabolism, Wnt/ Beta -high mutation rates of *AJUBA*, *MUC17*, *KMT2D*, and *NFE2L2*	Subtype 3 has the worst prognosis. Subtype 1 has the best prognosis. Subtype 1 has the most favorable response to immunotherapy.	Subtype 1 is mostly located in oropharynx (61%). Subtype 2 in oral cavity and Subtype 3 in larynx or hypopharynx.	171*
Gleber-Netto et al. (2019), JCI Insight	Discovery cohort: 80 OPSCC from TCGA (52 HPV+ and 28 HPV−). Validation cohort: 47 HPV16 + OPSCC from JHU and 138 HPV + CSCC from TCGA	All oropharynx	RNA Seq	RNA seq alignment	Pearson correlation to identify 582 HPV-correlated human transcript that were differentially expressed between HPV+ and HPV- OPSCC. Expression patterns of the 582 genes were used to divide HPV + OPSCC into C1 and C2. C1: mostly HPV+ cases. Exhibit intermediate gene expression of HPV-correlated genes. Statistically lower expression of HPV E1 and HPV E1^E4 gene. C2: all HPV+ cases. Exhibits high expression of HPV-correlated genes. C3: all HPV- cases. Exhibits low expression of HPV-correlated genes.	C2 has the best prognosis. C1 and C3 have similar prognosis.	No difference observed in HPV total or spliced gene expression between subgroups except E1 and E1^E4. E1^E4 expression correlated with cisplatin sensitivity in vitro. Differential HPV integration was not observed between C1 and C2.	114*
Locati (2019), Cancer	Discovery chort: Meta-analysis of 11 studies- total 346 HPV + HNSCC. Validation cohort: 47 HPV + OPSCC.	Discovery cohort: 235 oropharynx, 59 oral cavity, 20 larynx, 10 hypopharynx, others undetermined Validation cohort: All oropharynx	Microarray and RNA Seq	qPCR, HPV array, p16 IHC and RNAseq alignment	Cl1: Enrichment of immune components. Least percentage of HPV integration. Cl2: enrichment of keratinocytes and higher stromal score; high EMT score, highest percentage of HPV integration. Cl3: Enrichment of keratinocytes and lower stromal score; high proliferation.	Cl1 has the best prognosis, Cl2 has the worst prognosis. Cl1 has the higher percentage of early-stage tumors. Cl2 has the highest percentage of advanced-stage tumors.	C2 has the highest percentage of HNSCC that are not OPSCC.	[93]
Kim et al. (2020), British Journal of Cancer	37 surgically resected OPSCC (21 HPV + /p16 + , 1 HPV + /p16-, 1 HPV-/p16+, 14 HPV-/p16-). 9 pre-treated samples of recurrent or metastatic PD-1/PD-L1 treated OPSCC (HPV status not determined).	All oropharynx	RNA Seq	p16 IHC	IR: all HPV + . Enriched T cell exhaustion signature with PD-1 + CD8 + T cells and type I macrophages infiltrating the tumor nest, upregulation of APOBEC3B family genes and T-cell exhaustion genes. XB: all HPV-. scant CD8 + T cell infiltration and focal CD73 expression. Upregulation of XB metabolism genes and EMT. MS: mixed HPV status. exclusion of CD8 + T cells from the tumor nest and high MS and tumor growth factor-β, higer glycolytic activity signatures, enriched with genes related to smooth muscle contraction and cell adhesion and keratinization and EMT.	Among anti-PD-1/PD-L1-treated OPSCC, the IR subtype showed a favorable clinical response (3/4 patients), whereas the XB type showed early progression.	N.A	142*
Zhang et al. (2021), Frontiers	Total 944 HNSCC from 4 independent datasets TCGA HNSCC cohort (*n* = 546). Gene expression profiles from GSE107591 (*n* = 46), GSE127165 (*n* = 114), GSE41613 (*n* = 97), GSE65858 (*n* = 270) and GSE427433 (*n* = 75).	NIL	RNA Seq	HPV status was downloaded from Xena Public Data Hubs. However, it is not clarified if HPV status was determined by p16 testing or ISH.	C1: more HPV+ patients, least oncogenic pathway activation, immune strong, increased expression of immune checkpoint genes *(CD274, PDCD1 and CTLA4)*. C2: immune strong (highest stromal content), high oncogenic pathway activation; high tumor mutational burden. C3: high tumor mutational burden, low immune infiltration. Highest activation of UPR, mTORC signaling and UV response pathways.	C1 patients have the best overall survival (OS) and progession-free survival (PFS). C3 has the worst OS and PFS. C1 is predicted to have the highest response rate to immunotherapy and highest sensitivity to chemotherapy. C2 tumors are predicted to be responsive to HSP90 and MEK1/2 inhibitors. C3 tumors were predicted to be senstive to drugs that disrupt ER homeostasis and mTOR inhibitor.	Although most HPV+ patients are found in C1, there are some in C2 and C3.	154*
Zeng et al. (2022), EBioMedicine	863 HPV + OPSCC from 5 separate cohorts.	All oropharynx	RNA Seq	HPV genotyping via HPV transcript quantification.	Tumors are classified as immune-rich, immune desert or mixed based on an identified immune signature.	Immune-rich tumors have the best prognosis, while immune-desert tumors have the worst prognosis. Immune-rich tumors responded most favorably in phase II de-escalation trials, while immune-desert tumors are most likely to develop recurrence or metastasis.	N.A	153*

*ESCC* esophageal SCC, *LSCC* lung SCC, *CSCC* cervical SCC, *IHC* immunohistochemistry.

Given that most non-keratinizing OPSCC are HPV+, it is associated with favorable survival [78, 82, 83]. However, in a cohort of 208 p16+ patients, the non-keratinizing phenotype yielded better survival only in p16- but not in p16+ OPSCC. Within the p16+ sub-population, keratinization was only prognostic in smokers and not in non-smokers [83]. This suggests that morphologic subtype alone is an insufficient prognostic classifier for p16+ OPSCC.

While OPSCC can be grouped into distinct morphologic subtypes, it is important to discuss specific histologic characteristics that provide substantive prognostic information. One such feature is nuclear pleomorphism, such as anaplasia and multinucleation. Anaplasia is the presence of three or more cells with nuclei that have diameters of five or more lymphocyte nuclei, while multinucleation occurs when each tumor cell has three or more nuclei [82]. Anaplasia and multinucleation (A/M) increase from non-keratinizing to NKM to keratinizing subtypes [82]. The distribution of A/M also differs, with non-keratinizing OPSCC showing focal distribution, while the other subtypes likely present more diffuse distribution. A/M was significantly associated with worse disease-specific survival regardless of p16 status [82]; this was substantiated in multiple studies regardless of whether pathologists’ evaluation or computerized scoring was used [84–87]. As A/M reflects genomic instability, it likely highlights a subset of genetically complex OPSCC. However, the prognostic value of A/M in HPV + OPSCC has been challenged by other studies [88, 89], where A/M was not predictive of survival or recurrence. Therefore, A/M may be insufficient to predict prognosis of HPV + OPSCC.

With the rise of artificial intelligence, researchers are using machine-learning approaches to characterize histopathologic variables in HPV + OPSCC [84, 85, 90]. In one study, patients with poorer prognosis demonstrated greater inter- and intra-tumoral variations in nuclear morphologic parameters such as nuclear texture, granularity, and cytomorphology [90]. The molecular and biologic basis behind these divergent features both within and between tumors remains unclear. Understanding the interplay between molecular and morphologic phenotypes will help us understand how morphologic variations point toward distinct biological pathways that may influence treatment choices for subsets of HPV + OPSCC.

## Molecular heterogeneity of HPV+OPSCC

Heterogeneity in clinical and morphological profiles implies intrinsic heterogeneity that could be delineated by molecular profiling. Indeed, molecular characterization of HNSCC cohorts revealed distinct HPV+ subtypes (Table 2). Earlier molecular profiling studies such as The Cancer Genome Atlas (TCGA) did not distinguish OPSCC from non-OPSCCs [11, 91–93]; this is a major concern as OPSCC differs significantly from non-OPSCC. Consequently, non-OPSCC were overrepresented in the subtype with poorest prognosis. Nevertheless, these studies provide important clues into molecular heterogeneity of HPV + OPSCC, as most HPV + HNSCC are OPSCC. This could be validated by more recent studies that restrict analysis to HPV + OPSCC (Table 2).

Analyzing gene expression from key molecular characterization studies on HPV + HNSCC [11, 91–93], Qin et al. [94] noted substantial congruency and derived three main molecular subtypes of HPV + HNSCC. The first subtype is immune-enriched and associated with low HPV integration frequency and high mesenchymal differentiation, the second is highly keratinized and basal-like with high stromal content and metabolic signatures, and the third is a highly keratinized subtype with low stromal content and suppressed immune responses. Overall, molecular classification studies revealed variables associated with prognosis, including viral transcript expression and host genomic alterations. Importantly, a subgroup of HPV + OPSCC demonstrates aggressive clinical features and molecular profiles similar to HPV(-) counterparts, including poor clinical outcomes and treatment resistance (Table 2).

While HPV + OPSCC subtypes mentioned above reflect heterogeneity at inter-patient level, single-cell sequencing studies also revealed huge intratumoral heterogeneity within the same patient [95, 96]. Intratumoral heterogeneity is the phenomenon where different cell populations coexist in the same tumor; metastasis and treatment resistance may result when aggressive or persistor clones are selected over others. In HPV + OPSCC cancer cells, variations in chromosomal aberrations, pathway activation, and HPV viral gene transcript expression were reported between and within tumors with important prognostic implications [95]. Moreover, HPV + OPSCC also showed differing intratumoral inflammation and fibroblast elastic differentiation plasticity [96]. In the following sections, we discuss molecular variations that may contribute to inter- and intratumoral heterogeneity. This is important because molecular phenotyping illuminates more extensive information on tumoral heterogeneity than traditional histopathologic assessment, enabling robust stratification that more accurately reflects prognosis and treatment responses.

### HPV integration and extrachromosomal DNA

Since HPV is an etiologic factor for many OPSCCs variations in HPV biology likely affect clinical responses. Integration of HPV into the host genome is common in HPV+ cancers and could indicate poor prognosis [38]. RNA sequencing on HPV + HNSCC, 18 from University of Michigan and 66 from TCGA, of which 80% were OPSCC [92], identified two molecular subtypes, KRT and IMU. KRT has an increased keratinization signature, while IMU has a stronger immune response and mesenchymal signature [92]. Importantly, KRT OPSCCs are more aggressive than IMU and have significantly higher HPV integration frequency. Locati and colleagues published similar findings based on gene expression analysis in eleven cohorts [93]. They identified three distinct subtypes of HPV + HNSCC: Cl1, Cl2, and Cl3. The Cl1 corresponds to IMU with best survival and lowest HPV integration rates [93]. Positive correlation between HPV integration and poor prognosis were also reported by others [36, 37, 97, 98]; HPV integration-positive HNSCC had unfavorable survival similar to HPV(-) cancer [36]. Furthermore, HPV integration in HNSCC predicts aggressive clinical phenotypes such as large tumor size and perineural invasion [37]. However, other studies reported conflicting findings [35, 98–100]. Inconsistent results for the prognostic significance of HPV integration could be due to variations in detection methods. While some methods such as genome-wide sequencing detect integration events from genomic DNA, others such as PCR and RNA sequencing determine integration events through RNA transcripts [38]. These may affect the determination of HPV integration in tumor samples, as a small proportion of HPV-integrated tumors may lack active viral transcripts [23].

Various studies support that HPV integration in OPSCC is a nonrandom event, preferring genomic regions that harbor structural and copy number variants; this leads to dysregulated host gene expression near the integration site (35, 100, 101*, 102*). Many dysregulated genes, including *PD-L1*, *SOX2*, *TP63*, *FGFR3* and *MYC*, are involved in processes related to cancer progression such as immune cell function, epithelial differentiation, and proliferation (35, 36, 100, 101*, 102*). It is possible that the integration site determines OPSCC progression and therapy response. For example, Walline et al. (2016) showed that therapy-responsive OPSCC had HPV integration mostly at intergenic chromosome regions; in contrast, recurrent OPSCC were more likely to harbor integration at cancer-associated genes (103*). However, findings from this study were limited by small sample size (20 OPSCC) (103*). In cervical cancer, patients with “productive” HPV integration (i.e., generation of actively transcribed viral-host fusion transcript) have higher *E6/E7* expression, increased tumor aggressiveness, and immune evasion compared to those with “silent” integration (i.e., no viral-host fusion transcript) (104*). Similar studies on a large OPSCC cohort could determine the diverse consequences of HPV integration.

Genome-wide HPV integration drives pervasive instability that can affect OPSCC development and aggressiveness (35, 37, 100, 101*, 105*). Instability encourages host structural and copy number variations via genomic amplification, deletion, structural rearrangement, recombination, chromosomal translocations, and inversion (35, 37, 100, 101*, 105*). Using whole-genome sequencing in 105 OPSCC, Akagi et al. (2023) identified heterocateny as the predominant genomic structural alteration induced by HPV integration (102*). In heterocateny, numerous, diverse and repetitive virus and host DNA concatemers coexist within cancer cells leading to intratumoral heterogeneity. This promotes clonal evolution where tumor clones with genomic alterations promoting carcinogenesis are further amplified (102*).

Extrachromosomal DNA (ecDNA) is emerging as a critical driver of human cancers and could explain why some genes have high amplification rates (106*). Briefly, ecDNA is a circular DNA that exists outside chromosomes. Its circular structure and lack of centromeres allows ecDNA to be more accessible for transcription and promote unequal segregation into daughter cells, in turn driving oncogene transcription while enhancing intratumoral heterogeneity (107*). Fusion transcripts containing HPV *E6/E7* genes and host oncogenes were found in ecDNAs in OPSCC cell lines and patient samples, likely due to integration that promotes a highly unstable genome and results in multiple excision and reintegration events (102*, 108*, 109*). Consequently, virus-host transcripts found within ecDNA have structural variations and higher expression compared to those in intrachromosomal regions (102*, 109*). Therefore, ecDNA is another possible link between HPV integration and OPSCC progression. However, the precise roles of HPV-induced ecDNA amplification in cancer is unconfirmed.

In summary, HPV integration likely promotes OPSCC progression through three driver events: (i) dysregulation of oncogenes or tumor suppressor genes important for tumorigenesis, (ii) genome-wide alterations in distinct cancer subclones to promote intratumoral heterogenenity, and (iii) ecDNA that promotes oncogene amplification and heterogeneity. Important questions remain: What are the biological and clinical implications of HPV integration-induced dysregulated gene expression and genome instability? When and how is HPV integration triggered? Can driver events that induce integration be counteracted or prevented? Development of HPV + OPSCC experimental models and accurate viral integration breakpoint prediction technologies will help evaluate the diagnostic, prognostic, and therapeutic significance of HPV integration in OPSCC. Furthermore, there is a need to determine whether tumors with HPV integration but minimal active viral transcript also share the same prognostic implications as those that harbor “silent” HPV integration [23].

### Variations in HPV transcript expression and functions

*E2* gene deletion and elevated *E6/E7* transcript levels may portend inferior clinical outcomes (110*, 111*). Higher *E6/E7* transcript and protein in serum of untreated patients also predicted higher risk of recurrence after treatment (112*, 113*). Other studies reported conflicting findings (114*, 115*). Comparing HPV gene expression in 84 patients with primary HPV16 + HNSCC, Zhang et al. (2016) observed no significant change in total *E6/E7* between KRT and IMU subtypes [92]. However, KRT with poorer clinical outcomes, has an increased ratio of alternatively spliced HPV16 E6 isoforms (E6*) relative to full-length expression [92]. In a follow-up study, HPV + OPSCC with high E6* activity scores are larger, have higher HPV integration rates, and poorer overall survival (116*). Furthermore, E6* scores were positively correlated with mutational burden, suggesting roles in DNA damage and radiation response (116*). Therefore, E6* may be important for progression and therapeutic response of OPSCC. However, the functional roles of E6* in OPSCC are still poorly understood. This is despite multiple reports that the spliced variant, E6*I is more abundantly transcribed in OPSCC than full-length E6 (103*, 109*, 117*), while another study showed almost equal E6 and E6*I in TCGA HNSCC samples (118*) Perhaps, the best characterized function of E6* is that it does not inhibit p53, unlike its full-length isoform (119*, 120*). Some studies suggested that alternative splicing of E6 into E6*I promotes efficient translation of E7 proteins (121*, 122*), but conflicting findings have been reported (123*, 124*). A major caveat is that most studies that interrogate the link between E6* and p53 were performed on cell lines originating from cervical cancer or other non-HPV related cancers (119*), while only one study used an overexpression approach in a HPV-negative OPSCC cell line that harbors p53 mutations (125*). There is a dire need to interrogate and validate functional and mechanistic roles of E6* isoforms using appropriate experimental models.

Another distinguishing molecular feature is that KRT has lower *E2/4/5* transcript levels than IMU [94]. This is consistent with a TCGA study that demonstrated two subgroups of HPV16 + HNSCC; one with high integration rate and increased *E6/E7* expression, and the other predominantly integration-negative with minimal *E6/E7*, but significant upregulation in *E2/4/5* [35]. A subsequent functional study suggested that *E2/4/5* expression drives alternative carcinogenic mechanisms independent of *E6/E7* (126*). Gleber-Netto et al. (2019) proposed two HPV16 + OPSCC subtypes that differed primarily by expression of 38 HPV16-correlated genes, and hence likely differed in HPV-modulated functions and biological pathways (114*). These two subtypes also vary significantly in survival, with HPV + C1 having poorer overall survival than HPV + C2. However, no significant differences in viral integration status or gene expression of HPV16 E2-E7 were observed between C1 and C2 (114*). In contrast, transcript expression of E1 and E1^E4 spliced isoforms was significantly higher in HPV + C2 than HPV + C1 (114*). In HPV16+ cell lines, elevated E1^E4 potentiated radiation sensitivity (114*). It is unclear if differential E1^E4 expression is due to HPV integration, as statistical association between integration and E1^E4 is weak (114*).

Puram et al. (2023), reported diverse intratumoral heterogeneity in HPV transcript expression in single-cell transcriptomic analysis of 12 HPV + OPSCC [95]. In one patient, malignant epithelial cells identified by chromosomal aberrations were observed in the tumor margin originally interpreted as histologically negative; these cells expressed higher *E5* transcript than those in the tumor bulk [95], suggesting that *E5* drives tumor invasion. There was also a subset of malignant cells within HPV+ tumors that had undetectable HPV expression (defined as HPV*off* cells), indicating that HPV transcripts were lost or reduced; such suppression may be driven by epigenetic regulation [95]. Importantly, these HPV*off* cells demonstrated phenotypes that are typically associated with HPV(-) OPSCC such as epithelial senescence, radiation resistance, and invasion [95]. Together, these findings support previous molecular characterization in bulk HPV+ tumors, where a proportion of HPV + OPSCC demonstrated aggressive phenotypes, similar to HPV(−) tumors.

Overall, inconsistencies between association of HPV transcripts and biologic functions in OPSCC reveal our lack of understanding of the roles of HPV proteins in carcinogenesis. It is possible that HPV transcript expression and/or activity should be considered together with other molecular factors such as HPV integration to predict clinical outcomes and therapy responses. The finding of HPV*off* cells in HPV + OPSCC also has potential clinical significance due to their reduced treatment responses and increased invasion in vitro. Future studies will need to understand how HPV*off* cells emerge in HPV + OPSCC, and their functional implications.

### HPV circulating tumor DNA

ctDNAs are short fragments of DNA released into systemic circulation during tumor cell apoptosis and/or necrosis. ctDNA detection represents a non-invasive approach to assess tumor burden dynamically (127*). HPV ctDNAs are detected in saliva and plasma in up to 95% of HPV + OPSCC using quantitative PCR, digital droplet PCR, and capture-based next-generation sequencing (127*, 128*). These assays commonly target high-risk HPV *E6/E7* oncogenes due to abundance in cancer patients (127*); in contrast, ctDNAs are undetectable in individuals with HPV infection but no cancer (128*).

Varying levels of HPV ctDNAs are observed in primary and metastatic OPSCC before, during, and after treatment. These variations have important diagnostic and prognostic implications. Elevated pre-treatment ctDNA correlated with higher overall disease burden (129*, 130*), larger tumor size (131*), and increased nodal burden (132*, 133*, 134*, 135*). In 110 primary OPSCC, patients with detectable pre-treatment ctDNA had higher clinical nodal stage, larger lymph nodes, and were more likely to present lymphovascular invasion (135*). In contrast, in a multi-institutional study, patients with more aggressive tumors (higher tumor and nodal stages, increased HPV16 integration) had low or undetectable pre-treatment HPV16 ctDNA (136*). The reasons behind these conflicting findings are unclear but could be due to differences in how HPV positivity was determined and whether recurrent tumors were included in the analysis. Low pre-treatment ctDNA may also predict recurrence after primary CRT, although this remains to be validated (133*, 136*). Together, these preliminary studies suggest that varying levels of pre-treatment HPV ctDNAs are related to stages of OPSCC progression including tumor growth, nodal metastasis, lymphovascular invasion, and HPV genomic integration. Future studies could explore if pre-treatment HPV ctDNA is a marker of aggressive tumors to inform therapy selection.

During treatment, HPV ctDNA levels change drastically due to increased tumor cell death (133*). Preliminary studies suggested that ctDNA levels peak from weeks 1–3 but clear by weeks 4–7 of standard CRT in primary OPSCC (131*, 133*, 136*). Kinetics of rise of HPV ctDNA and clearance correlate with therapeutic response (133*, 136*). In 103 non-metastatic p16+ OPSCC, patients who had >95% clearance of HPV ctDNA at Week 4 of CRT were likely to remain disease-free after treatment (136*). In 34 advanced p16+ OPSCC, an increase in HPV ctDNA levels at week 2 of CRT was associated with lower risk of disease progression but levels and clearance at weeks 4 or 7 did not predict progression (133*). Similar results were observed in patients with recurrent and metastatic tumors, where longitudinal changes in HPV ctDNA correlated with treatment response (137*, 138*). Successful remission should be reflected by complete clearance of HPV ctDNA in both primary and metastatic disease (131*, 136*). However, HPV ctDNA persists in some patients even after treatment, indicating residual disease (139*, 140*); elevation post-therapy is predictive of recurrence (140*).

Collectively, there is wide inter-patient variability in HPV ctDNA levels and kinetics before, during, and post-treatment. It is unclear if these variations are reflected in HPV+ molecular subtypes, such as KRT and IMU. Nevertheless, ease and convenience to obtain and analyze HPV ctDNA makes it an attractive candidate for real-time disease monitoring. Moreover, changes likely occur even before anatomic or radiologic response (131*, 140*), enabling timelier intervention (i.e., adaptation of ongoing treatment and initiation of salvage therapy) to improve treatment outcomes.

### Variations in other biologic processes

Based on microarray and RNA sequencing studies (Table 2), distinct subgroups of HPV + OPSCC frequently exhibit differential enrichment in keratinization, metabolism, and immune responses. As mentioned in section “Morphologic heterogeneity of HPV+ OPSCC”, HPV + OPSCC mostly presents a non-keratinizing morphology, although a small proportion demonstrates focal or diffuse keratinization. It is unclear whether keratinization in HPV + OPSCC truly portends worse survival. However, elevated expression of keratin 17 has been correlated with poor prognosis in OPSCC regardless of HPV status (141*). Moreover, several omics analyses proposed that elevated expression of keratinization genes predicts inferior survival (92, 142*). Whether keratin gene expression truly reflects histopathology and aggressiveness requires further investigation.

Using whole exome sequencing to compare the mutational landscape, Harbison and colleagues reported that primary HPV + OPSCC that recurred after treatment had mutations in genes involved in metabolic and oxidative stress responses compared to non-recurrent tumors (143*). Expression of an oxidative metabolic gene signature comprising 200 genes negatively correlated with overall survival in three separate multi-institutional HPV + OPSCC cohorts (144*). Based on the TCGA cohort and preliminary experiments on HPV + OPSCC cell lines, increasing levels of full-length E6, but not E6*, mitigated mitochondrial anti-oxidant capacity and promoted cisplatin sensitivity (144*). Conversely, previous studies reported an important role of HPV16 E6*, not full-length E6, in inducing oxidative stress and modulating mitochondrial function in cervical and oropharyngeal cancers (116*, 145*, 146*). Together, these studies support an essential role of oxidative stress and mitochondrial metabolism in driving HPV + OPSCC progression. Other processes such as glucose metabolism and hypoxia are also associated with prognosis (147*, 148*, 149*). Glucose- and hypoxia-based imaging markers predict survival and recurrence in HPV + OPSCC (148*, 150*), although controversy remains (151*, 152*).

Molecular profiling of HPV + OPSCC consistently demonstrated that subtypes with better prognosis were enriched in immune-related processes (92, 93, 142*, 153*, 154*). Tumors that do not harbor HPV integration have higher lymphocytic infiltration and stronger immune signatures characterized by enrichment in T (CD3 + , CD4 + , CD8 + ), B, NK, and CD34^+^ cells [36]. In agreement, tumor-infiltrating lymphocytes (TILs) have been associated with improved prognosis in HPV + OPSCC (155*, 156*, 157*, 158*). T-cell immunity may promote survival in HPV + OPSCC by inducing a T_H_1 anti-tumor response (155*, 159*). CD163+ dendritic cells are key players that drive tumor-specific T cell infiltration and Th1 polarization (159*). Moreover, HPV proteins such as E2/E5/E6/E7 promote immunoevasion that contributes to malignant transformation and therapy resistance through various mechanisms; these include suppression of MHC class molecules, inhibition of stimulator of interferon genes (STING) activation, modulation of CD4+ and regulatory T-cell function, and regulation of the PD-1/PD-L1 immune checkpoint (160*). Therefore, targeting of HPV proteins such as E5, E6 and E7 is a potential therapeutic strategy to improve sensitivity to anti–PD-1/PD-L1 immunotherapy (161*).

While most studies focus on the role of T-cell immunity in HPV + OPSCC, HPV-specific B cells also provide prognostic information (162*, 163*, 164*, 165*, 166*). In a cohort of 72 OPSCC, higher infiltration of CD20 + B cells and B cell/CD8 + T cell interactions predicted superior prognosis, surpassing HPV status and T-cell infiltration (162*). In another study, increased abundance of peri-tumoral B cells in the lymph nodes correlates with better survival (167*). Additionally, cancer-associated fibroblasts with elastic differentiation negatively predict overall survival [96]. Analysis of immune genes in the TCGA HNSCC cohort revealed that SYNGR3, a neuronal gene expressed in T and B cells, was overexpressed in HPV + HNSCC compared to HPV(-) HNSCC. This was validated in multiple cohorts. Enhanced SYNGR3 expression correlates with improved survival in HPV + HNSCC (168*). Together, the immune environment clearly modulates HPV + OPSCC progression; however, whether and how each immune component regulates HPV-driven tumor development in the oropharynx requires further investigation. In summary, heterogeneity in processes such as immune activation and hypoxia reflects the biology of HPV + OPSCC. Variations in biologic processes provide valuable clues to biomarkers that may refine selection for de-intensification clinical trials. In a Phase 2 trial, patients who responded well to de-intensified 30 Gy compared to the original 70 Gy had either no pre-treatment hypoxia or resolution of hypoxia during treatment (148*). Concurrently, Zeng et al. (2022) proposed a classification where immune-rich patients classified by a three-gene immune classifier had excellent survival outcomes and were more likely to remain disease-free even after receiving de-escalated RT (30 Gy) (153*). Other studies identified rapid alterations in immune cell composition and transcriptomes during early treatment stage (169*, 170*), suggesting that molecular markers can predict mid-treatment response to assist precise adaptive treatment selection. Immune biomarkers have the potential to predict responses to immunotherapies as well. Currently, there is evidence showing that high PD-L1 expression correlates with improved response to PD-L1/PD-1 inhibitor in recurrent and metastatic patients [53]. In locally advanced patients, clinical benefit for PD-L1/PD-1 inhibitor is uncertain due to high variability in response among high-PD-L1 expressors [54].

As mentioned in section “Clinical Management of HPV-associated OPSCC”, HPV E6/E7 vaccines and T-cell therapy are emerging immunotherapeutics against HPV + OPSCC. Therefore, biomarkers that predict response to these therapies have not been discovered. Nevertheless, selecting HPV + OPSCC patients suitable for immunotherapy remains a top priority. Future studies should aim to uncover novel markers that can predict response to immunotherapies especially in the upfront definitive setting to aid selection for treatment de-escalation.

### Genomic and epigenomic alterations

Various genomic and epigenomic alterations have been identified among HPV+ subtypes. *E2F3* amplification is higher in classical than inflamed/mesenchymal subtypes, and coupled with overexpression of cell cycle genes including *MCM2, MCM10, CDKN2A, E2F2*, and *RPA2* [91]*. Zhang et al. (2016), observed that KRT has increased frequency of PIK3CA activating mutations and copy number gains, as well as amplifications at* chr3q [92]. IMU harbored frequent copy number loss on chr16q, which was absent in HPV-KRT and HPV(-) samples [92]. Lee et al. (2018) observed differential *PIK3CA* and *NOTCH* mutations between subtypes (171*). *PIK3CA* is the most commonly mutated gene; ~20–30% of HPV + OPSCC carry this mutation (11, 117*, 172*). Mutations of phosphoionositide 3-kinase (PI3K) components and downstream mediators have been associated with prognosis and treatment response in HPV + OPSCC (173*). Patients with mutant rather than wild-type *PIK3CA* are more likely to develop recurrence three years after completing de-intensified CRT (174*). Therefore, patients with wild-type *PIK3CA* would be better candidates for de-escalated therapy in primary HPV + OPSCC.

The TCGA head and neck cancer cohorts were classified into different subgroups based on DNA methylation profile [11]. HPV + HNSCC samples were distributed across all subgroups, supporting heterogeneity in global DNA methylation [11]. The subgroup that is hypomethylated is associated with higher non-synonymous mutations, reduced EMT signature, and superior overall survival [11]. In another study on a TCGA HNSCC cohort, tumors with HPV integration had similar methylation patterns as HPV(-) HNSCC and normal tissues, whereas HNSCC with episomal HPV (integration-negative) had different methylation profiles from the other three groups. These findings substantiate the proposition that HPV-integrated HNSCC has distinct carcinogenic mechanisms from those with episomal HPV [35]. It is also of interest to explore if methylation patterns differ based on HPV+ molecular subtypes. Using the University of Michigan HNSCC cohort, Liu and colleagues (175*) characterized 5-hydroxymethylation (5hmC) profiles in the KRT and IMU subtypes identified previously [92]. Using 5hmC profiles only, less aggressive IMU could be easily distinguished from KRT, which had 5hmC profiles similar to those of HPV(-) HNSCC. Closer annotation of 5hmC profiles revealed hyper-5hmC and increased expression of cell migration genes in IMU. In contrast, KRT had hyper-5hmC enrichment related to keratinization and cell junctions, with accompanied elevation in gene expression.

The apolipoprotein B mRNA editing enzyme catalytic polypeptide-like (APOBEC) cytidine deaminases is a family of enzymes that convert cytosine to uracil in nucleotides. HPV + OPSCC is highly enriched with a *APOBEC m*utagenesis signature that strongly correlates with mutational burden (117*, 176*, 177*). Overexpression of APOBEC3A *(*A3A*)*, A3B, A3F and A3H were detected in HPV-infected keratinocytes and OPSCC compared to their HPV(-) counterparts, due to dysregulation by E6 and E7 oncoproteins (178*, 179*, 180*, 181*). Mutations induced by APOBEC can occur simultaneously in viral and host genomes (182*); key somatic mutations include driver mutations in the *PIK3CA* helical domain and other immune genes (176*, 183*). In another study, A3A promoted HPV16 E2 hypermutation and associated with increased viral genome integration in OPSCC (184*). While these findings suggest that APOBEC mutagenesis signature portends poorer prognosis, another study contradicts by demonstrating that A3 enzymes improve survival and response to cisplatin in HPV + HNSCC (185*). Future studies will need to confirm the prognostic impact of APOBEC mutagenesis signature in HPV + OPSCC. It will also be interesting to investigate if distinct molecular subtypes of HPV + OPSCC express differential APOBEC-induced mutational pattern and load.

Overall, molecular characterization of HPV + OPSCC revealed diverse inter- and intra-tumoral levels; some studies proposed molecular subtypes but it is unclear how these subtypes affect treatment selection. This could be due to lack of prospective studies that associate treatment outcomes with molecular subtypes in HPV + OPSCC. A web-based tool that can differentiate the molecular subtypes of HPV + OPSCC will be useful for researchers to classify their samples and associate them with important clinical variables.

## Challenges and future directions

Despite the suggestion of several biomarkers from patient and/or experimental studies, none is currently available to classify HPV + OPSCC into high and low risk groups for de-escalated therapy. One key reason could be lack of substantial validation, due to conflicting results, possibly from small population size or inconsistencies in selection criteria for clinical trials. Future studies that include several hundred patients (up to thousands) with corresponding data from molecular assays, imaging, and clinical outcomes could help to identify and validate key biomarkers with substantial clinical implications. Another major caveat is that many biomarkers are identified from bulk tumor populations whereas spatial and temporal changes related to tumor progression may yield more predictive results. Delineating the main factors that drive heterogeneity in HPV + OPSCC will help to identify candidates for de-intensified therapy and personalized treatment strategies.

To better understand the biology of HPV + OPSCC, in vitro and in vivo models that can recapitulate the heterogeneity in HPV + OPSCC are needed. However, HPV + HNSCC cell lines harbor multiple genetic artifacts, which are misrepresented in patient specimens. These artifacts include overrepresentation of *EGFR* amplification and underrepresentation of canonical *PIK3CA*-activating mutations and *TRAF3* (186*). Furthermore, many HPV + HNSCC cell lines are poorly engrafted in immune-deficient mice (186*, 187*) and only a few HPV+ oropharyngeal cell lines exist (186*). Patient-derived xenografts or organoids could overcome these shortcomings to improve capture of heterogeneity and architecture (187*, 188*). However, success rates for engrafting HPV + OPSCC into xenograft or organoids are low (187*), and most do not include the tumor microenvironment, which is important for progression (189*). Novel ex vivo tumor organoids that incorporate tumor microenvironment characteristics are under development and require validation (190*). There is a pressing need for models that recapitulate genetic variation, tumor morphologies, and architectures within the tumor microenvironment in distinct HPV + OPSCC subgroups.

Recent studies revealed that race/ethnicity-specific differences may impact incidence, survival, and morphology of cancers (191*). In HNSCC, patients with European ancestry (White) significantly outnumber those of African ancestry (Black) (10, 85, 192*, 193*). White patients are more likely to present HPV + HNSCC (192*, 193*). In contrast, Black patients are usually diagnosed at a younger age and have poorer survival, even after accounting for HPV status, cancer stage, and healthcare access (192*, 194*). The reason behind this racial disparity is unclear, but may be due to differing environmental factors, diet, and genetic variations (192*, 194*). A multi-institutional study comprising 744 HPV + OPSCC suggested that Black patients have a higher density of multinucleated tumor cells than White patients, independent of AJCC-8 stage [85]. This suggests that certain prognostic biomarkers for HPV + OPSCC should be population-specific. Other studies revealed variations in immune, mutational and transcriptomic profiles among HNSCC in White versus Black patients (192*, 195*, 196*). However, whether these findings translate to HPV + OPSCC remains unknown. Unraveling the characteristics of HPV + OPSCC in different racial and ethnic subgroups may provide insights into biologic factors underlying their prognostic variations.

## Conclusion

**Fig. 3:**
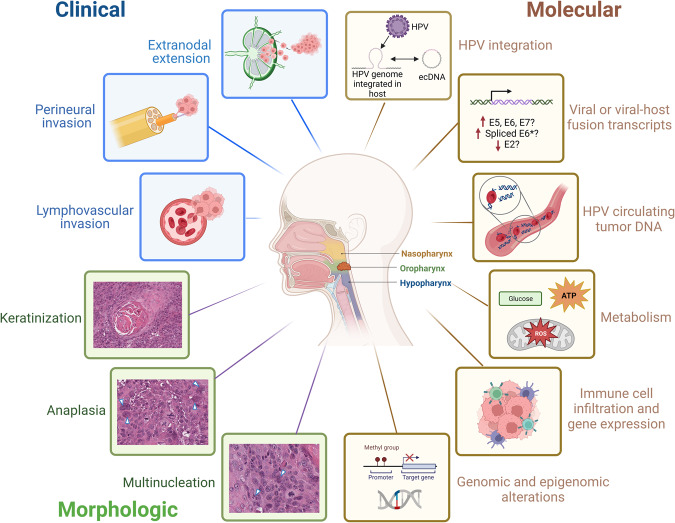
Clinical, morphologic, and molecular features associated with aggressive HPV + OPSCC. Clinical features are illustrated in blue, morphologic in green and molecular in brown boxes. Hematoxylin-eosin (H&E) images were used to illustrate keratinization, anaplasia and multinucleation in HPV + OPSCC tumors. Arrows point to the morphologic feature described. This figure was created using BioRender.

Recognition of HPV + OPSCC with its favorable clinical outcomes sparked interest in de-escalating treatment to reduce side effects. However, inter- and intratumoral heterogeneity are a monumental challenge. Biomarkers are important for assigning patients to accurate risk subgroups. In this review, we discussed potential biomarkers based on clinical, histopathologic, and molecular variations (Fig. 3). It is likely that molecular features correspond to clinical and morphologic profiles but their links remain largely unexplored. There is a need to integrate clinical, histopathologic, and molecular variations to develop a robust and clinically actionable paradigm that groups HPV + OPSCC into subtypes. Multidisciplinary collaboration is needed to accurately integrate the plethora of markers and determine the most robust and reliable biomarker combination for routine diagnosis. A more detailed understanding of tumor development, evolution and resistance will provide clinically-relevant information to improve outcomes for all HPV + OPSCC; this can be achieved through appropriate experimental models that better represent heterogeneity in HPV + OPSCC.

### Supplementary information

Supplementary Table 1

Supplementary references
