# Supplementary material for: Clinical, morphologic and molecular heterogeneity of HPV-associated oropharyngeal cancer
Source: Oncogene. 2023 Sep 4;42(40):2939–55. doi: 10.1038/s41388-023-02819-y (PMC10541327; doi:10.1038/s41388-023-02819-y)
Supplement: Supplementary file 1 — Supplementary Table 1 [file 41388_2023_2819_MOESM1_ESM.docx]

**Supplementary Table 1:** List of completed or ongoing clinical de-intensification trials

| **De-intensification Approach** | **Study** | **Number of patients** | **Treatment** | **Status/ findings** | **Refs** |
| --- | --- | --- | --- | --- | --- |
| (1) Substituting cisplatin with cetuximab or immunotherapy | RTOG 1016 (Phase II randomized) | 849 locally advanced HPV+ OPSCC | Either RT plus cetuximab or RT plus cisplatin. RT was delivered at 70 Gy in 35 fractions over 6 weeks. | Completed. RT plus cetuximab showed poorer survival and similar toxicity compared to RT plus cisplatin. | (1) |
|  | De-ESCALaTE (Phase III randomized | 334 locally advanced HPV+ OPSCC | Either RT plus cetuximab or RT plus cisplatin. RT was delivered at 70 Gy in 35 fractions over 6 weeks. | Completed. No differences in toxicity between the two treatment arms; cetuximab plus RT had poorer survival. | (2) |
|  | NCT03799445 (Phase II single arm) | Early-stage HPV+ OPSCC. (Estimated 180) | All given standard RT together with nivolumab and ipilimumab in place of cisplatin. | Ongoing | (3) |
|  | NCT03410615 (Phase II randomized) | 129 p16+ locoregionally advanced and non-metastastic OPSCC | Randomly assigned to: (A) standard RT+ concurrent cisplatin or (2) standard RT+ concurrent durvalumab followed by adjuvant durvalumab. | Ongoing | (4) |
|  | NCT01530997 and NCT02281955 (Phase II single arm) | 44 T1-T3, N0-N2c p16+ OPSCC | All given de-intensified RT of 60 Gy with weekly low-dose cisplatin. | Pathological complete response rate (ie absence of tumors during physical and radiological assessment) was high at 86% after de-intensfied treatment. Patients also had superior 3-year tumor control and survival with decreased acute and long-term toxicities. | (5) |
| (2) RT dose reduction to definitive or elective regions | ReACT or NCT04900623 (Phase II single arm) | Estimated 75 patients with non-metastatic HPV+ OPSCC | Stratified into high-risk or low-risk groups based on pretreatment levels of HPV circulating tumor DNA (ctDNA). High risk group will be given standard dose RT/CRT in 7-8 weeks. Low-risk will be given low dose RT/CRT in 5-6 weeks. ctDNA will be measured at mid-treatment (Week 4), end of treatment, and follow-up at 3, 6, 9 and 12 months after the treatment. | Ongoing | (6) |
|  | Tsai et al. (2022) (Phase II single arm) | 276 primary and untreated HPV+ OPSCC | Elective nodal regions were given reduced dose of 30 Gy, while gross lesions were treated with 70 Gy RT | High 2-year locoregional control of 97%, progression-free survival of 80%, distant metastasis-free survival of 95.2% and overall survival of 95.1%. Favorable quality of life profile. | (7) |
|  | EVADER or NCT03822897 (Phase II single arm) | 103 non-metastatic HPV+OPSCC | All given definitive standard CRT (70Gy) with reduced dose RT (56Gy) to some lymph nodes | Ongoing. | (8) (9) |
|  | ENID or NCT04444869 (Phase II single arm) | 28 with localized p16+ OPSCC | All given concurrent cisplatin and RT with reduced RT dose to clinically and radiologically uninvolved lymph nodes. | Ongoing | (10) |
|  | NRG Oncology HN002 or NCT02254278 (Phase II randomized) | 306 advanced HPV+ OPSCC | Arm 1: Reduced RT dose (60Gy) plus cisplatin. Arm 2: Reduced RT dose (60 Gy) without cisplatin. | Completed. 2-year locoregional failure rate was significantly higher in RT alone group. The grade 3–4 acute toxic effect was significantly higher in CRT group. | (11) |
|  | NCT03077243 (Phase II non-randomized | 215 non-metastatic HPV+ OPSCC | Arm 1: Patients with 10 or less pack years smoking history or >10 pack years, wild-type p53 receive de-intensified CRT of 60 Gy. Arm 2: Patients with > 10 pack years and p53 mutation receive standard CRT. | Ongoing. Changes in HPV ctDNA during and after treatment will be recorded | (12) |
|  | NCT03323463 (Phase II single arm) | 158 locoregional HPV+ OPSCC | Patients with no pre-RT hypoxia imaging were given de-intensified RT of 30Gy with concurrent CT. Remaining patients with pretreatment hypoxia received standard CRT (70 Gy) but underwent mid-treatment hypoxa imaging at 2 weeks of CRT. Patients with hypoxia resolution were given reduced CRT (30Gy) mid-treatment; rest continued to receive standard CRT | Completed. De-escalation to 30Gy using patient specific treatment response based on hypoxia resolution resulted in excellent locoregional control with significant toxicity reduction. Investigators will be validating this pilot study in a larger cohort. | (13) |
| (3) RT dose adaptation based on mid-treatment response | NCT03416153 (Phase II non-randomized) | Estimated 75 early stage HPV+ OPSCC | Initially standard RT. Dose subsequently reduced to 54Gy to high-risk planned target volume and 43.2Gy to low-risk planned target volume based on FDG-PET imaging | Ongoing | (14) |
|  | NCT03215719 (Phase II single-arm) | Estimated 54 early-stage HPV+ OPSCC | Initially standard CRT. At 4 weeks, patients get computer tomography scan. Patients with >40% nodal shrinkage will undergo de-escalated RT. | Ongoing | (15) |
|  | MR-ADAPTOR or NCT03224000 (Phase II randomized) | Estimated 75 p16+ non-metastatic OPSCC | Arm 1: Standard RT.Arm 2: magnetic resonance-guided RT | Ongoing | (16, 17) |
|  | NCT02048020 and NCT01716195 (Phase II single arm) | 44 locally advanced p16+ OPSCC | All received induction CT (paclitaxel and carboplatin) followed by risk-adapted de-intensified RT dose of 54 or 60 Gy | Completed. Patients have high progression-free survival and improved toxicity profile compared to historical regimens using standard doses. | (18) |
| (4) Induction CT followed by de-intensified and/or risk-adapted RT or CRT | E1308 (Phase II single arm) | 90 HPV+ localized HPV+ OPSCC | All patients received 3 cycles of induction CT. Patients with complete clinical response were given de-intensifed RT, while others were given standard RT with concurrent cetuximab. | Completed. Reduced dose CRT in favorable and low-risk patients conferred favoral survival and quality of life profiles | (19) |
|  | OPTIMA (Phase II non-randomized) | 62 HPV+ OPSCC patients. 28 patients were considered as low-risk (≤T3, ≤N2B, ≤10 pack-year history) and 34 patients were classified as high-risk (T4 or ≥N2C or >10 pack-year history) | All patients received 3 cycles of induction CT (carboplatin/nab-paclitaxel). Response assessed by Response Evaluation Criteria in Solid Tumors 1.1. Low-risk patients with ≥50% response after induction CT received 50 Gy RT (RT50) while low-risk patients with 30%–50% response or high-risk patients with ≥50% response received 45 Gy RT and concurrent cisplatin. All other patients received standard RT of 75 Gy and concurrent cisplatin | Completed. Induction chemotherapy with response and risk-stratified dose and volume de-escalated RT/CRT for HPV+ OPSCC is associated with favorable oncologic outcomes and reduced acute and chronic toxicity. | (20) |
|  | Quarterback trial or NCT01706939 (Phase III randomized) | 20 locally advanced HPV+ OPSCC | All received 3 cycles of induction CT and then randomized to standard CRT (70Gy) or de-intensified CRT (56Gy) | Completed. Standard CRT or de-intensified CRT demonstrated similar overall- and progression-free survival. However, patients who received de-intensified CRT had less toxic effects 5 years after treatment | (21) |
| (4) Transoral surgery followed by de-escalated and risk-dependent adjuvant therapy | ORATOR (Phase II randomized) | 68 early-stage OPSCC (60 HPV+) | Evenly randomized into (A) standard RT or (B) transoral surgery with neck dissection. | Completed. Transoral surgery with neck dissection did not yield superior quality of life compared to standard RT | (22) |
|  | ORATOR-2 (Phase II randomized) | 61 early-stage p16+ OPSCC | De-intensified CRT (primary RT of 60Gy plus cisplatin) vs transoral surgery with adjuvant RT | Excessive toxicity in transoral surgery arm | (23) |
|  | PATHOS or NCT02215265 (Phase II/III randomized) | Estimated 1100 non-metastatic HPV+ OPSCC | All given transoral surgery then stratified into low-, intermediate-, or high-risk based on pathologic features (tumor stages, lymph node involvement, perineural invasion, vascular invasion or extranodal extension).  Low-risk with no adverse pathologic features not given adjuvant treatment. Intermediate-risk randomized into either adjuvant RT of 50 or 60Gy.  High-risk randomized into 60 Gy RT alone or with concurrent cisplatin. | Ongoing | (24) |
|  | IIT2019-20-Zumsteg-HPVOPC or NCT04502407 (Phase II single-arm) | Estimated 36 HPV+ OPSCC with no distal metastasis and recurrence | Resection via transoral robotic surgery then classified into high-risk (positive margins, extranodal extension or 5 or more lymph nodes) or low-risk. High-risk given CRT with reduced dose of 50 Gy. Low-risk given CRT of 30Gy. | Ongoing. | (25) |
|  | SIRS or NCT02072148 (Phase II non-randomized) | 54 with previously untreated p16+ OPSCC and | All given transoral surgery and stratified into low-, intermediate- and high-risk. Low-risk had no poor risk features post-surgery and not given adjuvant therapy. Intermediate-risk had complete resection but demonstrated perineural invasion, lymphovascular invasion and <3 positive lymph nodes; given adjuvant de-intensified CRT of 50Gy. High-risk had >3 lymph nodes and positive margins; given post-operative 50 or 56 Gy CRT. | Completed. High disease-specific (98.1%) and progression-free survival (90.7%) after a median follow-up of 43.9 months. | (26) |
|  | SIRS2.0 or NCT05419089 (Phase II non-randomized) | Estimated 199 p16+ OPSCC | All treated with transoral surgery and then stratified into high- and low-risk based on pathological features. Also undergo blood test to determine presence of HPV ctDNA after surgery. Low-risk pathologic disease (negative margins, <4 nodes, and no gross extranodal extension) and no detectable HPV ctDNA not given adjuvant therapy. High-risk pathologic disease and undetectable post-operative HPV ctDNA receive de-intensifed adjuvant CRT (46Gy) | Ongoing | (27) |
|  | NCT05387915 (Phase II single group assignment) | Estimated 33 HPV+ early-stage OPSCC | All receive transoral surgery then tested for HPV ctDNA. Patients negative for HPV ctDNA given reduced dose standard RT for 3 weeks; patients with detectable HPV ctDNA undergo standard RT. | Ongoing | (28) |
|  | ADAPT or NCT03875716 (Phase II non-randomized) | Estimated 111 early-stage HPV+ OPSCC | All undergo curative-intent surgery. Patients with 2 or less positive lymph nodes with no extranodal extension and negative margins after surgery not given adjuvant therapy; others given reduced dose of 46 Gy or 60 Gy RT (no CT) based on pathological assessment. | Ongoing | (29) |
|  | AVOID or NCT02159703 (single arm Phase II) | 60 with lymph node involvement | Transoral robotic surgery combined with de-intensified RT given as adjuvant therapy (54Gy to uninvolved neck regions and 60-66Gy to involved neck areas). Patients with extranodal extension given concurrent CT. | Completed. Favorable survival and toxicity outcomes. 2-year local recurrence-free survival was 97.9%. OS was 100%. Only 2 of 60 patients had soft tissue necrosis. | (30) |
|  | E3311 (Phase II randomized) | 445 Stage III-IVA p16+ OPSCC who underwent transoral robotic surgery | Postoperative RT was assigned based on risk criteria. Low-risk with no extranodal extension not given adjuvant therapy. Intermediate-risk with 1mm or less ENE or four positive nodes randomly assigned to 50Gy or 60Gy postoperative RT. High-risk (>1mm ENE or 5 or more metastatic lymph nodes) received 66 Gy postoperative CRT. | Completed. Favorable survival and functional outcomes reported in intermediate-risk HPV+ OPSCC. 2-year progression-free survival was 96.9%. | (31) |

**References**

1. Gillison ML, Trotti AM, Harris J, Eisbruch A, Harari PM, Adelstein DJ, et al. Radiotherapy plus cetuximab or cisplatin in human papillomavirus-positive oropharyngeal cancer (NRG Oncology RTOG 1016): a randomised, multicentre, non-inferiority trial. The Lancet. 2019;393(10166):40-50.

2. Mehanna H, Robinson M, Hartley A, Kong A, Foran B, Fulton-Lieuw T, et al. Radiotherapy plus cisplatin or cetuximab in low-risk human papillomavirus-positive oropharyngeal cancer (De-ESCALaTE HPV): an open-label randomised controlled phase 3 trial. The Lancet. 2019;393(10166):51-60.

3. Ipilimumab, Nivolumab, and Radiation Therapy in Treating Patients With HPV Positive Advanced Oropharyngeal Squamous Cell Carcinoma: ClinicalTrials.gov

[Available from: <https://clinicaltrials.gov/ct2/show/NCT03799445>.

4. Cisplatin + Radiotherapy vs Durvalumab + Radiotherapy Followed by Durvalumab vs Durvalumab + Radiotherapy Followed by Tremelimumab + Durvalumab in Intermediate-Risk HPV-Positive Oropharyngeal SCC: ClinicalTrials.gov; [Available from: <https://clinicaltrials.gov/ct2/show/NCT03410615>.

5. Chera BS, Amdur RJ, Tepper J, Qaqish B, Green R, Aumer SL, et al. Phase 2 Trial of De-intensified Chemoradiation Therapy for Favorable-Risk Human Papillomavirus–Associated Oropharyngeal Squamous Cell Carcinoma. International Journal of Radiation Oncology*Biology*Physics. 2015;93(5):976-85.

6. Risk-adapted Therapy in HPV+ Oropharyngeal Cancer Using Circulating Tumor (ct)HPV DNA Profile - The ReACT Study: ClinicalTrials.gov; [Available from: <https://clinicaltrials.gov/ct2/show/NCT04900623>.

7. Tsai CJ, McBride SM, Riaz N, Kang JJ, Spielsinger DJ, Waldenberg T, et al. Evaluation of Substantial Reduction in Elective Radiotherapy Dose and Field in Patients With Human Papillomavirus–Associated Oropharyngeal Carcinoma Treated With Definitive Chemoradiotherapy. JAMA Oncology. 2022;8(3):364-72.

8. De-Escalation Radiotherapy in Patients With Low-Risk HPV-Related Oropharyngeal Squamous Cell Carcinoma (EVADER): ClinicalTrials.gov; [Available from: <https://clinicaltrials.gov/ct2/show/NCT03822897>.

9. Bratman SV, Berthelet E, Butler JB, de Almeida JR, Karam I, Metser U, et al. CCTG HN.10: A phase II single-arm trial of elective volume adjusted de-escalation radiotherapy (EVADER) in patients with low-risk HPV-related oropharyngeal squamous cell carcinoma (NCT03822897). Journal of Clinical Oncology. 2020;38(15_suppl):TPS6592-TPS.

10. Testing Less Intensive Radiation With Chemotherapy to Treat Low-risk Patients With HPV-positive Oropharyngeal Cancer (ENID): ClinicalTrials.gov; [Available from: <https://clinicaltrials.gov/ct2/show/NCT04444869>.

11. Yom SS, Torres-Saavedra P, Caudell JJ, Waldron JN, Gillison ML, Xia P, et al. Reduced-Dose Radiation Therapy for HPV-Associated Oropharyngeal Carcinoma (NRG Oncology HN002). Journal of Clinical Oncology. 2021;39(9):956-65.

12. P53 Mutational Status and cf HPV DNA for the Management of HPV-associated OPSCC: ClinicalTrials.gov; [Available from: <https://clinicaltrials.gov/ct2/show/NCT03077243>.

13. Lee NY, Sherman EJ, Schöder H, McBride SM, Yu Y, Kang J, et al. The 30 ROC trial: Precision intra-treatment imaging guiding major radiation reduction in human papillomavirus related oropharyngeal cancer. Journal of Clinical Oncology. 2021;39(15_suppl):6019-.

14. Individualized Adaptive De-escalated Radiotherapy for HPV-related Oropharynx Cancer: ClinicalTrials.gov; [Available from: <https://clinicaltrials.gov/ct2/show/NCT03416153>.

15. Adaptive Treatment De-escalation in Favorable Risk HPV-Positive Oropharyngeal Carcinoma: ClinicalTrials.gov; [Available from: <https://clinicaltrials.gov/ct2/show/NCT03215719>.

16. Bahig H, Yuan Y, Mohamed ASR, Brock KK, Ng SP, Wang J, et al. Magnetic Resonance-based Response Assessment and Dose Adaptation in Human Papilloma Virus Positive Tumors of the Oropharynx treated with Radiotherapy (MR-ADAPTOR): An R-IDEAL stage 2a-2b/Bayesian phase II trial. Clinical and Translational Radiation Oncology. 2018;13:19-23.

17. Trial of Magnetic Resonance Imaging Guided Radiotherapy Dose Adaptation in Human Papilloma Virus Positive Oropharyngeal Cancer: ClinicalTrials.gov; [Available from: <https://clinicaltrials.gov/ct2/show/NCT03224000>.

18. Chen AM, Felix C, Wang P-C, Hsu S, Basehart V, Garst J, et al. Reduced-dose radiotherapy for human papillomavirus-associated squamous-cell carcinoma of the oropharynx: a single-arm, phase 2 study. The Lancet Oncology. 2017;18(6):803-11.

19. Marur S, Li S, Cmelak AJ, Gillison ML, Zhao WJ, Ferris RL, et al. E1308: Phase II Trial of Induction Chemotherapy Followed by Reduced-Dose Radiation and Weekly Cetuximab in Patients With HPV-Associated Resectable Squamous Cell Carcinoma of the Oropharynx— ECOG-ACRIN Cancer Research Group. Journal of Clinical Oncology. 2016;35(5):490-7.

20. Seiwert TY, Foster CC, Blair EA, Karrison TG, Agrawal N, Melotek JM, et al. OPTIMA: a phase II dose and volume de-escalation trial for human papillomavirus-positive oropharyngeal cancer. Annals of Oncology. 2019;30(2):297-302.

21. Takahashi M, Hwang M, Misiukiewicz K, Gupta V, Miles BA, Bakst R, et al. Quality of Life Analysis of HPV-Positive Oropharyngeal Cancer Patients in a Randomized Trial of Reduced-Dose Versus Standard Chemoradiotherapy: 5-Year Follow-Up. Frontiers in Oncology. 2022;12.

22. Nichols AC, Theurer J, Prisman E, Read N, Berthelet E, Tran E, et al. Radiotherapy versus transoral robotic surgery and neck dissection for oropharyngeal squamous cell carcinoma (ORATOR): an open-label, phase 2, randomised trial. The Lancet Oncology. 2019;20(10):1349-59.

23. Palma DA, Prisman E, Berthelet E, Tran E, Hamilton S, Wu J, et al. Assessment of Toxic Effects and Survival in Treatment Deescalation With Radiotherapy vs Transoral Surgery for HPV-Associated Oropharyngeal Squamous Cell Carcinoma: The ORATOR2 Phase 2 Randomized Clinical Trial. JAMA Oncology. 2022;8(6):845-51.

24. Owadally W, Hurt C, Timmins H, Parsons E, Townsend S, Patterson J, et al. PATHOS: a phase II/III trial of risk-stratified, reduced intensity adjuvant treatment in patients undergoing transoral surgery for Human papillomavirus (HPV) positive oropharyngeal cancer. BMC Cancer. 2015;15(1):602.

25. Trial of De-Intensified Post-operative Chemoradiation Following Robotic Surgery for HPV-positive Oropharyngeal Cancer: ClinicalTrials.gov; [Available from: <https://clinicaltrials.gov/ct2/show/NCT04502407>.

26. Miles BA, Posner MR, Gupta V, Teng MS, Bakst RL, Yao M, et al. De-Escalated Adjuvant Therapy After Transoral Robotic Surgery for Human Papillomavirus-Related Oropharyngeal Carcinoma: The Sinai Robotic Surgery (SIRS) Trial. The Oncologist. 2021;26(6):504-13.

27. The Sinai Robotic Surgery Trial in HPV-related Oropharyngeal Squamous Cell Carcinoma (SIRS 2.0 Trial): ClinicalTrials.gov; [Available from: <https://clinicaltrials.gov/ct2/show/NCT05419089>.

28. Biomarker-Driven Radiation Therapy Dose Reduction After Transoral Robotic Surgery for the Treatment of HPV-Positive Oropharyngeal Cancer: ClinicalTrials.gov; [Available from: <https://www.clinicaltrials.gov/ct2/show/NCT05387915>.

29. Study of De_Intensified Postoperative Radiation Therapy for HPV Associated Oropharyngeal Squamous Cell Carcinoma: ClinicalTrials.gov; [Available from: <https://clinicaltrials.gov/ct2/show/NCT03875716>.

30. Swisher-McClure S, Lukens JN, Aggarwal C, Ahn P, Basu D, Bauml JM, et al. A Phase 2 Trial of Alternative Volumes of Oropharyngeal Irradiation for De-intensification (AVOID): Omission of the Resected Primary Tumor Bed After Transoral Robotic Surgery for Human Papilloma Virus–Related Squamous Cell Carcinoma of the Oropharynx. International Journal of Radiation Oncology*Biology*Physics. 2020;106(4):725-32.

31. Ferris RL, Flamand Y, Weinstein GS, Li S, Quon H, Mehra R, et al. Phase II Randomized Trial of Transoral Surgery and Low-Dose Intensity Modulated Radiation Therapy in Resectable p16+ Locally Advanced Oropharynx Cancer: An ECOG-ACRIN Cancer Research Group Trial (E3311). Journal of Clinical Oncology. 2022;40(2):138-49.
