# Supplementary material for: Clinical, morphologic and molecular heterogeneity of HPV-associated oropharyngeal cancer
Source: Oncogene. 2023 Sep 4;42(40):2939–55. doi: 10.1038/s41388-023-02819-y (PMC10541327; doi:10.1038/s41388-023-02819-y)
Supplement: Supplementary file 2 — Supplementary references [file 41388_2023_2819_MOESM2_ESM.docx]

**Supplementary references**

**The following references are cited in the manuscript (text and tables) followed by an asterisk (*) and are not included in the list of references of the main text.**

101. Akagi K, Li J, Broutian TR, Padilla-Nash H, Xiao W, Jiang B, et al. Genome-wide analysis of HPV integration in human cancers reveals recurrent, focal genomic instability. Genome Research. 2014;24(2):185-99.

102. Akagi K, Symer DE, Mahmoud M, Jiang B, Goodwin S, Wangsa D, et al. Intratumoral heterogeneity and clonal evolution induced by HPV integration. Cancer Discovery. 2023.

103. Walline HM, Komarck CM, McHugh JB, Bellile EL, Brenner JC, Prince ME, et al. Genomic Integration of High-Risk HPV Alters Gene Expression in Oropharyngeal Squamous Cell Carcinoma. Molecular Cancer Research. 2016;14(10):941-52.

104. Fan J, Fu Y, Peng W, Li X, Shen Y, Guo E, et al. Multi-omics characterization of silent and productive HPV integration in cervical cancer. Cell Genomics. 2023;3(1):100211.

105. Gao G, Johnson SH, Vasmatzis G, Pauley CE, Tombers NM, Kasperbauer JL, et al. Common fragile sites (CFS) and extremely large CFS genes are targets for human papillomavirus integrations and chromosome rearrangements in oropharyngeal squamous cell carcinoma. Genes, Chromosomes and Cancer. 2017;56(1):59-74.

106. Wu S, Bafna V, Chang HY, Mischel PS. Extrachromosomal DNA: An Emerging Hallmark in Human Cancer. Annual Review of Pathology: Mechanisms of Disease. 2022;17(1):367-86.

107. Zhu Y, Gujar AD, Wong C-H, Tjong H, Ngan CY, Gong L, et al. Oncogenic extrachromosomal DNA functions as mobile enhancers to globally amplify chromosomal transcription. Cancer Cell. 2021;39(5):694-707.e7.

108. Deshpande V, Luebeck J, Nguyen N-PD, Bakhtiari M, Turner KM, Schwab R, et al. Exploring the landscape of focal amplifications in cancer using AmpliconArchitect. Nature Communications. 2019;10(1):392.

109. Pang J, Nguyen N, Luebeck J, Ball L, Finegersh A, Ren S, et al. Extrachromosomal DNA in HPV-Mediated Oropharyngeal Cancer Drives Diverse Oncogene Transcription. Clinical Cancer Research. 2021;27(24):6772-86.

110. Anayannis NV, Schlecht NF, Ben-Dayan M, Smith RV, Belbin TJ, Ow TJ, et al. Association of an intact E2 gene with higher HPV viral load, higher viral oncogene expression, and improved clinical outcome in HPV16 positive head and neck squamous cell carcinoma. PLOS ONE. 2018;13(2):e0191581.

111. Khwaja SS, Baker C, Haynes W, Spencer CR, Gay H, Thorstad W, et al. High E6 Gene Expression Predicts for Distant Metastasis and Poor Survival in Patients With HPV-Positive Oropharyngeal Squamous Cell Carcinoma. International Journal of Radiation Oncology*Biology*Physics. 2016;95(4):1132-41.

112. Oton-Gonzalez L, Rotondo JC, Lanzillotti C, Mazzoni E, Bononi I, Iaquinta MR, et al. Serum HPV16 E7 Oncoprotein Is a Recurrence Marker of Oropharyngeal Squamous Cell Carcinomas. Cancers. 2021;13(13):3370.

113. Fakhry C, Qualliotine JR, Zhang Z, Agrawal N, Gaykalova DA, Bishop JA, et al. Serum Antibodies to HPV16 Early Proteins Warrant Investigation as Potential Biomarkers for Risk Stratification and Recurrence of HPV-Associated Oropharyngeal Cancer. Cancer Prevention Research. 2016;9(2):135-41.

114. Gleber-Netto FO, Rao X, Guo T, Xi Y, Gao M, Shen L, et al. Variations in HPV function are associated with survival in squamous cell carcinoma. JCI Insight. 2019;4(1).

115. Spector ME, Sacco AG, Bellile E, Taylor JMG, Jones T, Sun K, et al. E6 and E7 Antibody Levels Are Potential Biomarkers of Recurrence in Patients with Advanced-Stage Human Papillomavirus–Positive Oropharyngeal Squamous Cell Carcinoma. Clinical Cancer Research. 2017;23(11):2723-9.

116. Qin T, Koneva LA, Liu Y, Zhang Y, Arthur AE, Zarins KR, et al. Significant association between host transcriptome-derived HPV oncogene E6* influence score and carcinogenic pathways, tumor size, and survival in head and neck cancer. Head & Neck. 2020;42(9):2375-89.

117. Gillison ML, Akagi K, Xiao W, Jiang B, Pickard RKL, Li J, et al. Human papillomavirus and the landscape of secondary genetic alterations in oral cancers. Genome Research. 2019;29(1):1-17.

118. Carper MB, Troutman S, Wagner BL, Byrd KM, Selitsky SR, Parag-Sharma K, et al. An Immunocompetent Mouse Model of HPV16(+) Head and Neck Squamous Cell Carcinoma. Cell Reports. 2019;29(6):1660-74.e7.

119. Olmedo-Nieva L, Muñoz-Bello JO, Contreras-Paredes A, Lizano M. The Role of E6 Spliced Isoforms (E6*) in Human Papillomavirus-Induced Carcinogenesis. Viruses. 2018;10(1).

120. Pim D, Massimi P, Banks L. Alternatively spliced HPV-18 E6* protein inhibits E6 mediated degradation of p53 and suppresses transformed cell growth. Oncogene. 1997;15(3):257-64.

121. Tang S, Tao M, McCoy JP, Zheng Z-M. The E7 Oncoprotein Is Translated from Spliced E6*I Transcripts in High-Risk Human Papillomavirus Type 16- or Type 18-Positive Cervical Cancer Cell Lines via Translation Reinitiation. Journal of Virology. 2006;80(9):4249-63.

122. Ajiro M, Jia R, Zhang L, Liu X, Zheng Z-M. Intron Definition and a Branch Site Adenosine at nt 385 Control RNA Splicing of HPV16 E6*I and E7 Expression. PLOS ONE. 2012;7(10):e46412.

123. del Moral-Hernández O, López-Urrutia E, Bonilla-Moreno R, Martínez-Salazar M, Arechaga-Ocampo E, Berumen J, et al. The HPV-16 E7 oncoprotein is expressed mainly from the unspliced E6/E7 transcript in cervical carcinoma C33-A cells. Archives of Virology. 2010;155(12):1959-70.

124. Stacey Simon N, Jordan D, Williamson Andrew JK, Brown M, Coote Joanna H, Arrand John R. Leaky Scanning Is the Predominant Mechanism for Translation of Human Papillomavirus Type 16 E7 Oncoprotein from E6/E7 Bicistronic mRNA. Journal of Virology. 2000;74(16):7284-97.

125. Pang E, Delic NC, Hong A, Zhang M, Rose BR, Lyons JG. Radiosensitization of Oropharyngeal Squamous Cell Carcinoma Cells by Human Papillomavirus 16 Oncoprotein E6∗I. International Journal of Radiation Oncology*Biology*Physics. 2011;79(3):860-5.

126. Ren S, Gaykalova DA, Guo T, Favorov AV, Fertig EJ, Tamayo P, et al. HPV E2, E4, E5 drive alternative carcinogenic pathways in HPV positive cancers. Oncogene. 2020;39(40):6327-39.

127. Haring CT, Dermody SM, Yalamanchi P, Kang SY, Old MO, Chad Brenner J, et al. The future of circulating tumor DNA as a biomarker in HPV related oropharyngeal squamous cell carcinoma. Oral Oncology. 2022;126:105776.

128. Jeannot E, Becette V, Campitelli M, Calméjane M-A, Lappartient E, Ruff E, et al. Circulating human papillomavirus DNA detected using droplet digital PCR in the serum of patients diagnosed with early stage human papillomavirus-associated invasive carcinoma. The Journal of Pathology: Clinical Research. 2016;2(4):201-9.

129. Hilke FJ, Muyas F, Admard J, Kootz B, Nann D, Welz S, et al. Dynamics of cell-free tumour DNA correlate with treatment response of head and neck cancer patients receiving radiochemotherapy. Radiotherapy and Oncology. 2020;151:182-9.

130. Hanna GJ, Supplee JG, Kuang Y, Mahmood U, Lau CJ, Haddad RI, et al. Plasma HPV cell-free DNA monitoring in advanced HPV-associated oropharyngeal cancer. Annals of Oncology. 2018;29(9):1980-6.

131. Haring CT, Brummel C, Bhambhani C, Jewell B, Neal MH, Bhangale A, et al. Implementation of human papillomavirus circulating tumor DNA to identify recurrence during treatment de-escalation. Oral Oncology. 2021;121:105332.

132. Dahlstrom KR, Li G, Hussey CS, Vo JT, Wei Q, Zhao C, et al. Circulating human papillomavirus DNA as a marker for disease extent and recurrence among patients with oropharyngeal cancer. Cancer. 2015;121(19):3455-64.

133. Cao Y, Haring CT, Brummel C, Bhambhani C, Aryal M, Lee C, et al. Early HPV ctDNA Kinetics and Imaging Biomarkers Predict Therapeutic Response in p16+ Oropharyngeal Squamous Cell Carcinoma. Clinical Cancer Research. 2022;28(2):350-9.

134. Cao H, Banh A, Kwok S, Shi X, Wu S, Krakow T, et al. Quantitation of Human Papillomavirus DNA in Plasma of Oropharyngeal Carcinoma Patients. International Journal of Radiation Oncology*Biology*Physics. 2012;82(3):e351-e8.

135. Rettig EM, Wang AA, Tran N-A, Carey E, Dey T, Schoenfeld JD, et al. Association of Pretreatment Circulating Tumor Tissue–Modified Viral HPV DNA With Clinicopathologic Factors in HPV-Positive Oropharyngeal Cancer. JAMA Otolaryngology–Head & Neck Surgery. 2022;148(12):1120-30.

136. Chera BS, Kumar S, Beaty BT, Marron D, Jefferys S, Green R, et al. Rapid Clearance Profile of Plasma Circulating Tumor HPV Type 16 DNA during Chemoradiotherapy Correlates with Disease Control in HPV-Associated Oropharyngeal Cancer. Clinical Cancer Research. 2019;25(15):4682-90.

137. Haring CT, Bhambhani C, Brummel C, Jewell B, Bellile E, Heft Neal ME, et al. Human papilloma virus circulating tumor DNA assay predicts treatment response in recurrent/metastatic head and neck squamous cell carcinoma. Oncotarget. 2021;12(13).

138. Lee JY, Garcia-Murillas I, Cutts RJ, De Castro DG, Grove L, Hurley T, et al. Predicting response to radical (chemo)radiotherapy with circulating HPV DNA in locally advanced head and neck squamous carcinoma. British Journal of Cancer. 2017;117(6):876-83.

139. Fakhry C, Blackford AL, Neuner G, Xiao W, Jiang B, Agrawal A, et al. Association of Oral Human Papillomavirus DNA Persistence With Cancer Progression After Primary Treatment for Oral Cavity and Oropharyngeal Squamous Cell Carcinoma. JAMA Oncology. 2019;5(7):985-92.

140. Chera BS, Kumar S, Shen C, Amdur R, Dagan R, Green R, et al. Plasma Circulating Tumor HPV DNA for the Surveillance of Cancer Recurrence in HPV-Associated Oropharyngeal Cancer. Journal of Clinical Oncology. 2020;38(10):1050-+.

141. Regenbogen E, Mo M, Romeiser J, Shroyer ALW, Escobar-Hoyos LF, Burke S, et al. Elevated expression of keratin 17 in oropharyngeal squamous cell carcinoma is associated with decreased survival. Head & Neck. 2018;40(8):1788-98.

142. Kim MH, Kim J-H, Lee JM, Choi JW, Jung D, Cho H, et al. Molecular subtypes of oropharyngeal cancer show distinct immune microenvironment related with immune checkpoint blockade response. British Journal of Cancer. 2020;122(11):1649-60.

143. Harbison RA, Kubik M, Konnick EQ, Zhang Q, Lee S-G, Park H, et al. The mutational landscape of recurrent versus nonrecurrent human papillomavirus–related oropharyngeal cancer. JCI Insight. 2018;3(14).

144. Sannigrahi MK, Rajagopalan P, Lai L, Liu X, Sahu V, Nakagawa H, et al. HPV E6 regulates therapy responses in oropharyngeal cancer by repressing the PGC-1α/ERRα axis. JCI Insight. 2022;7(18).

145. Williams VM, Filippova M, Filippov V, Payne KJ, Duerksen-Hughes P. Human Papillomavirus Type 16 E6* Induces Oxidative Stress and DNA Damage. Journal of Virology. 2014;88(12):6751-61.

146. Paget-Bailly P, Meznad K, Bruyère D, Perrard J, Herfs M, Jung AC, et al. Comparative RNA sequencing reveals that HPV16 E6 abrogates the effect of E6*I on ROS metabolism. Scientific Reports. 2019;9(1):5938.

147. Liu X, Liu P, Chernock RD, Kuhs KAL, Lewis JS, Jr., Luo J, et al. A prognostic gene expression signature for oropharyngeal squamous cell carcinoma. eBioMedicine. 2020;61.

148. Riaz N, Sherman E, Pei X, Schöder H, Grkovski M, Paudyal R, et al. Precision Radiotherapy: Reduction in Radiation for Oropharyngeal Cancer in the 30 ROC Trial. JNCI: Journal of the National Cancer Institute. 2021;113(6):742-51.

149. Nicolay NH, Wiedenmann N, Mix M, Weber WA, Werner M, Grosu AL, et al. Correlative analyses between tissue-based hypoxia biomarkers and hypoxia PET imaging in head and neck cancer patients during radiochemotherapy—results from a prospective trial. European Journal of Nuclear Medicine and Molecular Imaging. 2020;47(5):1046-55.

150. Cho H, Kim S, Jo K, Jeong YH, Kang WJ. Tumor Glucose Metabolism and Its Heterogeneity on F-18 FDG PET/CT Provide Better Prognostication in Nonmetastatic Human Papillomavirus-Related Oropharyngeal Squamous Cell Carcinoma. Cancers [Internet]. 2021; 13(21).

151. Gouw ZAR, La Fontaine MD, van Kranen S, van de Kamer JB, Vogel WV, van Werkhoven E, et al. The Prognostic Value of Baseline 18F-FDG PET/CT in Human Papillomavirus–Positive Versus Human Papillomavirus–Negative Patients With Oropharyngeal Cancer. Clinical Nuclear Medicine. 2019;44(5):e323-e8.

152. Moan JM, Amdal CD, Malinen E, Svestad JG, Bogsrud TV, Dale E. The prognostic role of 18F-fluorodeoxyglucose PET in head and neck cancer depends on HPV status. Radiotherapy and Oncology. 2019;140:54-61.

153. Zeng PYF, Cecchini MJ, Barrett JW, Shammas-Toma M, De Cecco L, Serafini MS, et al. Immune-based classification of HPV-associated oropharyngeal cancer with implications for biomarker-driven treatment de-intensification. eBioMedicine. 2022;86.

154. Zhang P, Li S, Zhang T, Cui F, Shi J-H, Zhao F, et al. Characterization of Molecular Subtypes in Head and Neck Squamous Cell Carcinoma With Distinct Prognosis and Treatment Responsiveness. Frontiers in Cell and Developmental Biology. 2021;9.

155. Welters MJP, Ma W, Santegoets SJAM, Goedemans R, Ehsan I, Jordanova ES, et al. Intratumoral HPV16-Specific T Cells Constitute a Type I–Oriented Tumor Microenvironment to Improve Survival in HPV16-Driven Oropharyngeal Cancer. Clinical Cancer Research. 2018;24(3):634-47.

156. Balermpas P, Rödel F, Rödel C, Krause M, Linge A, Lohaus F, et al. CD8+ tumour-infiltrating lymphocytes in relation to HPV status and clinical outcome in patients with head and neck cancer after postoperative chemoradiotherapy: A multicentre study of the German cancer consortium radiation oncology group (DKTK-ROG). Int J Cancer. 2016;138(1):171-81.

157. Harbison RA, Pandey R, Considine M, Leone RD, Murray-Stewart T, Erbe R, et al. Interrogation of T Cell–enriched Tumors Reveals Prognostic and Immunotherapeutic Implications of Polyamine Metabolism. Cancer Research Communications. 2022;2(7):639-52.

158. Masterson L, Lechner M, Loewenbein S, Mohammed H, Davies-Husband C, Fenton T, et al. CD8+ T cell response to human papillomavirus 16 E7 is able to predict survival outcome in oropharyngeal cancer. European Journal of Cancer. 2016;67:141-51.

159. Santegoets SJ, Duurland CL, Jordanova EJ, van Ham VJ, Ehsan I, Loof NM, et al. CD163+ cytokine-producing cDC2 stimulate intratumoral type 1 T cell responses in HPV16-induced oropharyngeal cancer. Journal for ImmunoTherapy of Cancer. 2020;8(2):e001053.

160. Shamseddine AA, Burman B, Lee NY, Zamarin D, Riaz N. Tumor Immunity and Immunotherapy for HPV-Related Cancers. Cancer Discovery. 2021;11(8):1896-912.

161. Miyauchi S, Sanders PD, Guram K, Kim SS, Paolini F, Venuti A, et al. HPV16 E5 Mediates Resistance to PD-L1 Blockade and Can Be Targeted with Rimantadine in Head and Neck Cancer. Cancer Research. 2020;80(4):732-46.

162. Hladíková K, Koucký V, Bouček J, Laco J, Grega M, Hodek M, et al. Tumor-infiltrating B cells affect the progression of oropharyngeal squamous cell carcinoma via cell-to-cell interactions with CD8+ T cells. Journal for ImmunoTherapy of Cancer. 2019;7(1):261.

163. Wieland A, Patel MR, Cardenas MA, Eberhardt CS, Hudson WH, Obeng RC, et al. Defining HPV-specific B cell responses in patients with head and neck cancer. Nature. 2021;597(7875):274-8.

164. Lechner A, Schlößer HA, Thelen M, Wennhold K, Rothschild SI, Gilles R, et al. Tumor-associated B cells and humoral immune response in head and neck squamous cell carcinoma. OncoImmunology. 2019;8(3):1535293.

165. Wood O, Woo J, Seumois G, Savelyeva N, McCann KJ, Singh D, et al. Gene expression analysis of TIL rich HPV-driven head and neck tumors reveals a distinct B-cell signature when compared to HPV independent tumors. Oncotarget. 2016;7(35).

166. Kim SS, Shen S, Miyauchi S, Sanders PD, Franiak-Pietryga I, Mell L, et al. B Cells Improve Overall Survival in HPV-Associated Squamous Cell Carcinomas and Are Activated by Radiation and PD-1 Blockade. Clinical Cancer Research. 2020;26(13):3345-59.

167. Pretscher D, Distel LV, Grabenbauer GG, Wittlinger M, Buettner M, Niedobitek G. Distribution of immune cells in head and neck cancer: CD8+ T-cells and CD20+B-cells in metastatic lymph nodes are associated with favourable outcome in patients with oro- and hypopharyngeal carcinoma. BMC Cancer. 2009;9(1):292.

168. Murphy RM, Tasoulas J, Porrello A, Carper MB, Tsai Y-H, Coffey AR, et al. Tumor Cell Extrinsic Synaptogyrin 3 Expression as a Diagnostic and Prognostic Biomarker in Head and Neck Cancer. Cancer Research Communications. 2022;2(9):987-1004.

169. Smith JD, Ludwig ML, Bhangale AD, Brummel C, Swiecicki PL, Worden FP, et al. Tumor immune microenvironment alterations using induction cetuximab in a phase II trial of deintensified therapy for p16-positive oropharynx cancer. Head & Neck.n/a(n/a).

170. Jie H-B, Srivastava RM, Argiris A, Bauman JE, Kane LP, Ferris RL. Increased PD-1+ and TIM-3+ TILs during Cetuximab Therapy Inversely Correlate with Response in Head and Neck Cancer Patients. Cancer Immunology Research. 2017;5(5):408-16.

171. Lee DJ, Eun Y-G, Rho YS, Kim EH, Yim SY, Kang SH, et al. Three distinct genomic subtypes of head and neck squamous cell carcinoma associated with clinical outcomes. Oral Oncology. 2018;85:44-51.

172. Nichols AC, Palma DA, Chow W, Tan S, Rajakumar C, Rizzo G, et al. High Frequency of Activating PIK3CA Mutations in Human Papillomavirus–Positive Oropharyngeal Cancer. JAMA Otolaryngology–Head & Neck Surgery. 2013;139(6):617-22.

173. Beaty BT, Moon DH, Shen CJ, Amdur RJ, Weiss J, Grilley-Olson J, et al. PIK3CA Mutation in HPV-Associated OPSCC Patients Receiving Deintensified Chemoradiation. JNCI: Journal of the National Cancer Institute. 2019;112(8):855-8.

174. Beaty BT, Moon DH, Shen CJ, Amdur RJ, Weiss J, Grilley-Olson J, et al. PIK3CA Mutation in HPV-Associated OPSCC Patients Receiving Deintensified Chemoradiation. JNCI: Journal of the National Cancer Institute. 2020;112(8):855-8.

175. Liu S, de Medeiros MC, Fernandez EM, Zarins KR, Cavalcante RG, Qin T, et al. 5-Hydroxymethylation highlights the heterogeneity in keratinization and cell junctions in head and neck cancers. Clinical Epigenetics. 2020;12(1):175.

176. Henderson S, Chakravarthy A, Su X, Boshoff C, Fenton Tim R. APOBEC-Mediated Cytosine Deamination Links PIK3CA Helical Domain Mutations to Human Papillomavirus-Driven Tumor Development. Cell Reports. 2014;7(6):1833-41.

177. Faden DL, Thomas S, Cantalupo PG, Agrawal N, Myers J, DeRisi J. Multi-modality analysis supports APOBEC as a major source of mutations in head and neck squamous cell carcinoma. Oral Oncology. 2017;74:8-14.

178. Warren CJ, Xu T, Guo K, Griffin LM, Westrich JA, Lee D, et al. APOBEC3A Functions as a Restriction Factor of Human Papillomavirus. Journal of Virology. 2015;89(1):688-702.

179. Mori S, Takeuchi T, Ishii Y, Yugawa T, Kiyono T, Nishina H, et al. Human Papillomavirus 16 E6 Upregulates APOBEC3B via the TEAD Transcription Factor. Journal of Virology. 2017;91(6):10.1128/jvi.02413-16.

180. Vieira VC, Leonard B, White EA, Starrett GJ, Temiz NA, Lorenz LD, et al. Human Papillomavirus E6 Triggers Upregulation of the Antiviral and Cancer Genomic DNA Deaminase APOBEC3B. mBio. 2014;5(6):10.1128/mbio.02234-14.

181. Westrich JA, Warren CJ, Klausner MJ, Guo K, Liu C-W, Santiago ML, et al. Human Papillomavirus 16 E7 Stabilizes APOBEC3A Protein by Inhibiting Cullin 2-Dependent Protein Degradation. Journal of Virology. 2018;92(7):10.1128/jvi.01318-17.

182. Faden DL, Kuhs KAL, Lin M, Langenbucher A, Pinheiro M, Yeager M, et al. APOBEC Mutagenesis Is Concordant between Tumor and Viral Genomes in HPV-Positive Head and Neck Squamous Cell Carcinoma. Viruses. 2021;13(8):1666.

183. Faden DL, Ding F, Lin Y, Zhai S, Kuo F, Chan TA, et al. APOBEC mutagenesis is tightly linked to the immune landscape and immunotherapy biomarkers in head and neck squamous cell carcinoma. Oral Oncology. 2019;96:140-7.

184. Kondo S, Wakae K, Wakisaka N, Nakanishi Y, Ishikawa K, Komori T, et al. APOBEC3A associates with human papillomavirus genome integration in oropharyngeal cancers. Oncogene. 2017;36(12):1687-97.

185. Conner KL, Shaik AN, Ekinci E, Kim S, Ruterbusch JJ, Cote ML, et al. HPV induction of APOBEC3 enzymes mediate overall survival and response to cisplatin in head and neck cancer. DNA Repair. 2020;87:102802.

186. Cheng H, Yang X, Si H, Saleh AD, Xiao W, Coupar J, et al. Genomic and Transcriptomic Characterization Links Cell Lines with Aggressive Head and Neck Cancers. Cell Reports. 2018;25(5):1332-45.e5.

187. Facompre ND, Sahu V, Montone KT, Harmeyer KM, Nakagawa H, Rustgi AK, et al. Barriers to generating PDX models of HPV-related head and neck cancer. The Laryngoscope. 2017;127(12):2777-83.

188. Basnayake BWMTJ, Leo P, Rao S, Vasani S, Kenny L, Haass NK, et al. Head and neck cancer patient-derived tumouroid cultures: opportunities and challenges. British Journal of Cancer. 2023.

189. LeSavage BL, Suhar RA, Broguiere N, Lutolf MP, Heilshorn SC. Next-generation cancer organoids. Nature Materials. 2022;21(2):143-59.

190. Jenkins RW, Aref AR, Lizotte PH, Ivanova E, Stinson S, Zhou CW, et al. Ex Vivo Profiling of PD-1 Blockade Using Organotypic Tumor Spheroids. Cancer Discovery. 2018;8(2):196-215.

191. Zavala VA, Bracci PM, Carethers JM, Carvajal-Carmona L, Coggins NB, Cruz-Correa MR, et al. Cancer health disparities in racial/ethnic minorities in the United States. British Journal of Cancer. 2021;124(2):315-32.

192. Mezghani N, Yao A, Vasilyeva D, Kaplan N, Shackelford A, Yoon A, et al. Molecular Subtypes of Head and Neck Cancer in Patients of African Ancestry. Clinical Cancer Research. 2023;29(5):910-20.

193. Chernock RD, Zhang Q, El-Mofty SK, Thorstad WL, Lewis JS, Jr. Human Papillomavirus–Related Squamous Cell Carcinoma of the Oropharynx: A Comparative Study in Whites and African Americans. Archives of Otolaryngology–Head & Neck Surgery. 2011;137(2):163-9.

194. O'Neill WQ, Wasman J, Thuener J, Chatfield-Reed K, Lukesic L, Kyasram R, et al. African Americans With p16+ and p16− Oropharyngeal Squamous Cell Carcinomas Have Distinctly Poor Treatment Outcomes Independent of Medical Care Access. JCO Oncology Practice. 2021;17(5):e695-e702.

195. Worsham MJ, Stephen JK, Lu M, Chen KM, Havard S, Shah V, et al. Disparate Molecular, Histopathology, and Clinical Factors in Head and Neck Squamous Cell Carcinoma Racial Groups. Otolaryngology–Head and Neck Surgery. 2012;147(2):281-8.

196. Yuan J, Hu Z, Mahal BA, Zhao SD, Kensler KH, Pi J, et al. Integrated Analysis of Genetic Ancestry and Genomic Alterations across Cancers. Cancer Cell. 2018;34(4):549-60.e9.
